# Geographic and population disparities in cutaneous melanoma in the United States: state-level trends and national population-level analyses

**DOI:** 10.1186/s12889-026-27396-z

**Published:** 2026-05-02

**Authors:** Biaoyou Chen, Yijing Tang, Jiajia Lin, Xian Wei, Zihao Wang, Han Wang, Duoping Wang

**Affiliations:** 1 Department of Otolaryngology - Head and Neck Surgery, Guangxi Medical University Cancer Hospital, 71 Hedi Road, Nanning, 530021 China; 2 Department of Breast, Bone & Soft Tissue Oncology, Guangxi Medical University Cancer Hospital, 71 Hedi Road, Nanning, 530021 China; 3 School of Public Health, Shantou University, Shantou, 515041 China; 4 Melanoma Diagnostic and Therapeutic Center, Guangxi Medical University Cancer Hospital, Nanning, 530021 China

**Keywords:** Incidence, Obesity, Cutaneous Melanoma, Ultraviolet Radiation

## Abstract

**Background:**

Over recent decades, cutaneous melanoma (CM) incidence in the United States (US) has increased but now appears to be plateauing, whereas mortality has declined. However, much less is known about how CM incidence and mortality vary between states, or about how differences between states in incidence relate to lifestyle factors, ambient UV exposure, and prevention or health care resources. At the population level, most studies have compared overall CM incidence and mortality across broad racial and ethnic groups without describing age-specific or state-specific patterns. This lack of granularity limits our understanding of when and where different population groups bear the greatest CM burden and where targeted prevention and early detection efforts are most needed.

**Methods:**

We conducted a cross-sectional study of the incidence and mortality trends of CM in the US from 2001 to 2019, including state-level analyses of temporal trends and ecological correlates, as well as national population-level analyses by age, sex, and race/ethnicity. Annual percent change (APC) and average annual percent change (AAPC) were calculated, and ecological correlations and multivariable linear regression models were fitted with state-level AAPC in CM incidence as the outcome.

**Results:**

Between 2001 and 2016, CM incidence increased significantly (APC, 1.95% [95% CI, 1.67% to 2.24%], *p* < 0.001) and then stabilized from 2016 to 2019 (APC, -0.59% [95% CI, -3.36% to 2.27%], *p* = 0.662). During the study period, mortality declined significantly (AAPC, -1.52% [95% CI, -2.38% to -0.66%], *p* < 0.001). A persistent upward incidence trend was observed in 24 states, predominantly in the Midwest and Southern regions. The incidence rate ratio (IRR) between the highest- and lowest-rate states increased from 2.63 in 2001 to 4.85 in 2019, whereas the mortality rate ratio (MRR) narrowed to 1.19 by 2019. In ecological analyses, higher state-level obesity prevalence and lower physical activity level were each associated with higher AAPC in CM incidence, whereas higher personal doctor rates were inversely associated. Average daily solar insolation and tanning/sunscreen policies were not significantly related to the AAPC. Across all racial/ethnic groups, CM incidence was higher in young women than in young men, with sex-specific crossover ages varying by race/ethnicity, while young men consistently experienced higher mortality. Age-stratified analyses showed that disparities in CM incidence and mortality rates between non-Hispanic Whites and other races/ethnicities were more pronounced in young individuals than in the elderly. There was also substantial interstate variation in incidence rate ratios comparing non-Hispanic Whites with other racial/ethnic groups.

**Conclusions:**

Despite overall plateauing incidence and declining mortality, CM remains highly unequal across states and population groups. Interstate incidence disparities widened over time, with marked variation by age, sex, and race/ethnicity. These findings provide a more detailed picture of geographic and population-level disparities in CM and may help inform future surveillance, risk stratification, and hypothesis-driven prevention research.

**Supplementary Information:**

The online version contains supplementary material available at 10.1186/s12889-026-27396-z.

## Background

Melanoma is among the most common cancers in the U.S. and remains a major public health burden [1]. Over recent decades, cutaneous melanoma (CM) incidence has risen steadily but has only recently begun to plateau [2]. However, mortality has declined following the widespread adoption of immune checkpoint inhibitors and BRAF/MEK–targeted therapies [2]. Similar temporal patterns have been consistently reported in national surveillance studies based on Surveillance, Epidemiology, and End Results Program (SEER) and the United States Cancer Statistics (USCS) data [3]. This issue is clinically and public health relevant because prevention opportunities, UV exposure environments, access to early detection, and health care resources vary widely across the US, potentially producing important differences in CM burden that are not captured by national estimates alone.

Geographically, CM has long been recognized as a cancer with marked spatial heterogeneity [4]. Incidence is substantially higher in regions with greater solar radiation [5] and in states with a predominance of non-Hispanic White (NHW) populations [6] and state-level incidence rates can differ by several-fold [7]. Some studies have examined state- or regional-level associations between UV exposure [8], latitude [5], sunbathing behavior [9], indoor tanning [10], related legislation [11], and CM incidence, but most were limited to single time points, selected states, or average rates rather than long-term trends [7, 12, 13]. Only a few studies in recent years have systematically compared all 50 states and the District of Columbia in terms of state-level differences in average annual percent change (AAPC). Even fewer have quantified whether interstate differences in incidence are widening over time while differences in mortality are narrowing. This gap is important because interstate variation in environmental exposure, demographic composition, behavioral risk factors, and access to health care may lead to substantial inequalities in CM burden that are not captured by national averages. Accordingly, a major aim of our study was to characterize interstate variation in CM incidence and mortality and to identify contextual factors associated with these disparities. Although all authors are based outside the United States, we selected the US as the study setting because it bears a substantial melanoma burden, shows marked interstate and population-level heterogeneity, and provides nationally comprehensive public databases for transparent and reproducible analysis. As a multidisciplinary team involved in melanoma-related clinical care and research, we considered this setting particularly informative for understanding inequities in melanoma burden and generating insights relevant to cancer prevention and health equity. Beyond UV-related factors, other state-level structural determinants such as obesity prevalence and physical activity also vary widely across the United States (US) and may contribute to differences in CM incidence trends. The US has experienced a sustained growth in obesity rates in recent times [14]. The correlation between obesity and an increased risk of several cancers has been proven [15]. The association between obesity and CM incidence is currently a matter of dispute [16, 17]. Several meta-analyses, however, have shown that higher BMI is associated with an increased risk of CM among men, whereas no clear association has been observed in women [15, 16, 18]. Therefore, we also examined whether obesity prevalence, physical activity, average daily solar insolation, indoor tanning and school sunscreen policies, and health care access were associated with interstate variation in CM incidence trends by fitting multivariable ecological models with state-level AAPC in CM incidence as the outcome.

At the population level, CM incidence and mortality are substantially higher in white populations than in other racial/ethnic groups [19]. Among younger adults, incidence is higher in women than in men [20], whereas mortality is higher in men than in women [21]. Prior work has attributed these sex differences to a combination of biological and behavioral factors (e.g., hormonal factors, skin phototype, time spent outdoors, and indoor tanning) [22], and has documented racial/ethnic inequities in stage at diagnosis and survival [23]. However, most population-level analyses have used broad age categories and have not described in detail the ages at which male and female incidence or mortality curves cross, nor have they systematically examined age-specific IRRs and MRRs comparing NHWs with non-NHW groups across the life course and across states. These gaps limit a more precise understanding of when and in which populations CM burden becomes greatest, thereby constraining risk stratification and targeted prevention efforts. Accordingly, in this study we jointly analyzed CM incidence and mortality by age, sex, and race/ethnicity and described these patterns in more detail across the life course. Our aim was to provide more fine-grained evidence to guide targeted prevention and early detection strategies for different population groups at different stages of life.

## Methods

This study was designed as a state-level ecological analysis using aggregated incidence, mortality, and covariate data across US states. Population-based cancer incidence and mortality data by state, sex, races/ethnicities, and age were obtained from the USCS of the CDC WONDER online databases. USCS combines data from the National Program of Cancer Registries and the SEER Program and covers nearly 100% of the US population, including all 50 states and the District of Columbia [24]. The International Classification of Diseases for Oncology, 3rd edition (ICD-O-3) was used to identify the CM cases. The ICD-O-3 has been the standard reference for coding the histology of tumors diagnosed in 2001 and thereafter. Therefore, we selected the time from 2001 to 2019 as the study period (site: C44.0–44.9; histology: 8720–8790) [25]. We did not include data for diagnosis year 2020 because the COVID-19 pandemic disrupted cancer screening, diagnosis, and reporting in many regions, which may have affected the completeness and comparability of cancer statistics [24].

In this study, policy-related covariates were obtained from the National Environmental Public Health Tracking Network (NEPHTN). The NEPHTN, a web-based surveillance system with components at the national, state, and local levels, houses environmental public health data used to inform public health actions to improve community health [26]. The NEPHTN provided us with detailed data regarding legal restrictions on indoor tanning for minors and laws allowing students to carry and self-apply sunscreen in schools. In addition, we obtained long-term average daily solar insolation (kWh/m^2^/day) from the NASA satellite–based dataset available through the CDC WONDER interface. This measure was used as a proxy for UV exposure at the state level in the multivariable ecological analyses.

Behavioral and healthcare-related covariates were derived from the Behavioral Risk Factor Surveillance System (BRFSS), a nationwide, random-digit-dialed telephone survey for the US adult population [27]. Data on state-level obesity and physical activity were obtained from BRFSS. obesity was defined as individuals with body mass index (BMI) ≥ 30 kg/m2. Physical activity was defined as individuals who participated in 150 min or more of aerobic physical activity per week. For the multivariable models, we additionally obtained state-level estimates of health care coverage (having any kind of health care coverage: yes/no) and the proportion of respondents reporting that they have at least one person they think of as their personal doctor or health care provider (“personal doctor rate”), both derived from BRFSS. The state-level proportion of NHWs was obtained from the 2010 US Census and included as a covariate in the multivariable analyses.

This study used only publicly available, aggregate state-level data, with no access to any personal or case-level information. Therefore, institutional review board approval and informed consent were not required.

### Statistical analysis

Incidence and mortality rates and 95% CI were age-standardized to the 2000 US standard population and expressed per 100,000 population. The annual percent change (APC) and AAPC in CM incidence and mortality rates were estimated using the NCI’s Joinpoint Regression Program (version 4.9.1.0). This software used the best-fitting log-linear regression model to identify the calendar years when APCs changed significantly. The model was tested starting with the minimum number of joinpoints (i.e. no joinpoints), and examined whether more joinpoints are statistically significant [28]. IRR and MRR were calculated to quantify the magnitude of the differences in the incidence and mortality rates by races/ethnicities. For interstate comparisons, IRRs and MRRs were calculated as the ratios of the highest to the lowest state-specific age-adjusted incidence and mortality rates, respectively, within the same calendar year. For race/ethnicity comparisons, they were calculated by dividing the rate in NHWs by the corresponding rate in each comparison racial/ethnic group. Age-specific rates were used for age-stratified analyses, and age-adjusted rates were used for state-level race/ethnicity analyses. Pearson correlation analysis was used to assess the ecological correlation between state-level AAPC and obesity rateand physical activity level.

For the ecological analyses, we fitted multivariable linear regression models with state-level AAPC in CM incidence as the dependent variable and a set of state-level sociodemographic, behavioral, environmental, ethnic/racial, and policy covariates as independent variables. Model assumptions were evaluated using standard diagnostic procedures, and multicollinearity was assessed with variance inflation factors (VIFs), which were all < 5, supporting the use of ordinary least squares regression. Because the unit of analysis was the state, we additionally assessed global spatial autocorrelation of the regression residuals using Moran’s I with a first-order contiguity weights matrix; the absence of significant residual spatial autocorrelation indicated that spatial regression models were not required.

R software (version 4.0.4) and GraphPad Prism 8.0.2 were used to visualize the data. Two-sided *P* < 0.05 showed that the differences were statistically significant.

## Results

### CM incidence, mortality, and trends by overall, sex, age, and races/ethnicities

A total of 1,303,136 incident CM cases and 161,565 CM deaths were recorded between 2001 and 2019. The Age-adjusted incidence rate of CM was 20.67 per 100,000 Person-Years (PY) during study period (95% CI 20.64–20.71). The Age-adjusted CM mortality rate was 2.53 per 100,000 PY (95% CI 2.52–2.54). The incidence of CM increased with an AAPC of 1.53%/yr (*P* < 0.001) during 2001–2019. Joinpoint analyses showed that incidence increased by 1.95%/yr (*P* < 0.001) from 2001 to 2016. From 2016 to 2019, CM incidence was statistically stable, with no significant increase or decrease (− 0.59% per year; *p* = 0.66; Supplementary Table 1). Consistent with recent advances in melanoma therapy, the overall mortality rate declined, with an APC of 6.44% per year (*P* < 0.001) during 2013–2017, and then plateaued thereafter (Supplementary Table 1).

### Geographic variations in state-level CM incidence, mortality, and trends

In recent years, overall CM incidence in the US has begun to plateau, but state-level incidence trends still varied considerably across the country. Joinpoint Regression analyses showed that incidence continued to increase in 24 states, remained plateaued in 22 states, and decreased significantly in 5 states in recent years. Among the 24 states with increasing trends, the largest concentrations were in the Midwest and South, 11 states were in the Midwest region, including Illinois, Iowa, Kansas, Michigan, Minnesota, Missouri, Nebraska, North Dakota, Ohio, South Dakota, and Wisconsin, and 7 states were in the South region, including Arkansas, Kentucky, Louisiana, Mississippi, North Carolina, Virginia, and West Virginia; the remaining states were distributed across other U.S. regions (Table 1).

**Table 1:** Age-adjusted CM incidence rates and joinpoint trends, 2001–2019, by states

	Incidence rates per 100,000 PY			No of new cases	Incidence rates per 100,000 PY (95%CI)	Trend1/Trend3			Trend2/Trend4			2001–2019	
States	2001	2016	2019	2001–2019	2001–2019	Years	APC (95%CI)	*P* values	Years	APC (95%CI)	*P* value	AAPC	*P* value
1. Rising trends in recent years													
Arizona	19.97 (18.78,21.22)	26.84(25.71,28.02)	26.79(25.71,27.9)	28,453	21.52 (21.27,21.78)	2001–2007	−5.13* (−9.93,−0.09)	0.047	2007–2019	6.02* (4.5,7.56)	< 0.001	2.16* (0.33,4.03)	0.02
Arkansas	13.71 (12.37,15.16)	22.44 (20.85,24.13)	27.14 (25.41,28.95)	11,036	18.03 (17.69,18.38)	2001–2019	4.73* (4.09,5.38)	< 0.001				4.73* (4.09,5.38)	< 0.001
California	19.27 (18.79,19.77)	23.1 (22.64,23.58)	23.1 (22.65,23.56)	153,251	21.6 (21.49,21.71)	2001–2019	1.19* (0.9,1.48)	< 0.001				1.19* (0.9,1.48)	< 0.001
Hawaii	15.34 (13.27,17.65)	25.94 (23.49,28.57)	26.52 (24.08,29.13)	6,183	20.87 (20.35,21.41)	2001–2019	1.91* (0.98,2.84)	< 0.001				1.91* (0.98,2.84)	< 0.001
Idaho	19.82 (17.41,22.46)	30.52 (27.97,33.24)	29.92 (27.52,32.47)	8,013	26.44 (25.85,27.03)	2001–2019	1.74* (1.12,2.36)	< 0.001				1.74* (1.12,2.36)	< 0.001
Illinois	13.32 (12.68,13.98)	22.3 (21.52,23.1)	24.06 (23.26,24.88)	46,811	18.3 (18.13,18.47)	2001–2008	4.58* (2.97,6.22)	< 0.001	2008–2013	0.89 (−2.32,4.22)	0.558	3.53* (2.43,4.64)	< 0.001
						2013–2019	4.54* (2.92,6.17)	< 0.001					
Iowa	19.62 (18.08,21.26)	29.49 (27.67,31.4)	32.01 (30.14,33.95)	15,452	23.98 (23.6,24.37)	2001–2003	−4.66 (−17.48,10.16)	0.491	2003–2019	3.74* (3.24,4.24)	< 0.001	2.77* (1.22,4.34)	< 0.001
Kansas	17.4 (15.86,19.05)	27.71 (25.86,29.66)	29.01 (27.14,30.97)	13,663	23.92 (23.52,24.33)	2001–2019	2.59* (2.14,3.05)	< 0.001				2.59* (2.14,3.05)	< 0.001
Kentucky	22.23 (20.81,23.71)	29.19 (27.68,30.76)	27.84 (26.39,29.34)	22,098	24.75 (24.42,25.08)	2001–2019	2.04* (1.63,2.45)	< 0.001				2.04* (1.63,2.45)	< 0.001
Louisiana	11.04 (10.07,12.08)	16.59 (15.46,17.79)	18.29 (17.12,19.51)	13,865	15.45 (15.19,15.71)	2001–2019	3.06* (2.43,3.7)	< 0.001				3.06* (2.43,3.7)	< 0.001
Maine	21.1 (18.75,23.67)	28.26 (25.71,31)	24.85 (22.54,27.36)	7,499	24.14 (23.58,24.71)	2001–2019	1.43* (0.89,1.98)	< 0.001				1.43* (0.89,1.98)	< 0.001
Maryland	19.78 (18.59,21.01)	24.12 (22.94,25.35)	26.71 (25.51,27.97)	26,110	22.29 (22.02,22.57)	2001–2019	1.47* (0.96,1.98)	< 0.001				1.47* (0.96,1.98)	< 0.001
Michigan	18.8 (17.96,19.68)	21.37 (20.52,22.26)	19.29 (18.49,20.12)	40,339	19.39 (19.2,19.58)	2001–2019	0.62* (0.16,1.07)	0.011				0.62* (0.16,1.07)	0.011
Minnesota	18.8 (17.61,20.06)	32.2 (30.77,33.69)	35.46 (33.99,36.98)	29,449	27.29 (26.98,27.61)	2001–2019	4.38* (3.87,4.89)	< 0.001				4.38* (3.87,4.89)	< 0.001
Mississippi^a^		18.35 (16.89,19.91)	19.58 (18.08,21.18)	9,054	16.96 (16.61,17.32)	2003–2019	1.93* (1.19,2.67)	< 0.001				1.93* (1.19,2.67)	< 0.001
Montana	17.51 (14.95,20.39)	27.84 (24.86,31.08)	30.5 (27.45,33.79)	5,206	23.95 (23.29,24.63)	2001–2019	3.59* (3.05,4.13)	< 0.001				3.59* (3.05,4.13)	< 0.001
Nevada^b^	18.15 (16.31,20.13)	18.94 (17.45,20.51)		7,528	16.49 (16.11,16.87)	2001–2015	−1.82* (−2.82,−0.82)	0.002	2015–2017	29.62* (9.59,53.32)	0.006	1.65 (−0.42,3.75)	0.119
North Carolina	13.92 (13.12,14.75)	26.45 (25.5,27.43)	27.49 (26.56,28.45)	44,489	23 (22.79,23.22)	2001–2008	6.69* (3.98,9.46)	< 0.001	2008–2019	1.39* (0.41,2.38)	0.009	3.42* (2.33,4.52)	< 0.001
Ohio	15.96 (15.24,16.7)	26.5 (25.62,27.41)	26.72 (25.85,27.61)	51,628	21.13 (20.95,21.32)	2001–2019	2.65* (2.05,3.25)	< 0.001				2.65* (2.05,3.25)	< 0.001
South Dakota	12.43 (10.08,15.16)	25.43 (22.19,29.01)	23.18 (20.26,26.43)	3,542	20.85 (20.15,21.56)	2001–2019	4.13* (3.04,5.24)	< 0.001				4.13* (3.04,5.24)	< 0.001
Virginia	14.7 (13.81,15.62)	19.66 (18.76,20.59)	21.86 (20.94,22.81)	31,565	19.68 (19.46,19.9)	2001–2007	7.16* (3.65,10.78)	0.001	2007–2010	−7.6 (−22.86,10.69)	0.356	2.06 (−0.89,5.11)	0.173
						2010–2019	2.13* (0.57,3.72)	0.012					
Washington	23.11 (21.89,24.38)	25.59 (24.47,26.74)	27.66 (26.54,28.81)	35,139	25.81 (25.54,26.09)	2001–2019	0.55* (0.19,0.93)	0.006				0.55* (0.19,0.93)	0.006
West Virginia	17.21 (15.43,19.14)	22.19 (20.24,24.28)	21.85 (19.91,23.94)	8,532	20.39 (19.95,20.84)	2001–2019	1.64* (1.08,2.2)	< 0.001				1.64* (1.08,2.2)	< 0.001
Wyoming	22.7 (18.71,27.31)	20.04 (16.73,23.84)	26.54 (22.69,30.88)	2,542	22.62 (21.73,23.54)	2001–2019	1.52* (0.27,2.78)	0.02				1.52* (0.27,2.78)	0.02
2. No significant change in recent years													
Alabama	13.8 (12.74,14.92)	24.56 (23.25,25.92)	19.7 (18.56,20.9)	19,729	19.83 (19.55,20.11)	2001–2008	7.2* (3.04,11.53)	0.002	2008–2019	0.46 (−1.16,2.1)	0.553	3.03* (1.32,4.77)	< 0.001
Alaska	14.71 (11.18,18.97)	14.95 (12.02,18.37)	15.31 (12.47,18.58)	1,614	13.51 (12.82,14.23)	2001–2019	1.2 (−0.23,2.65)	0.096				1.2 (−0.23,2.65)	0.096
Colorado	20.45 (19.07,21.89)	22.74 (21.51,24.03)	21.19 (20.05,22.38)	21,344	22.08 (21.78,22.38)	2001–2019	0.28 (−0.17,0.74)	0.206				0.28 (−0.17,0.74)	0.206
Delaware	20.92 (17.89,24.31)	29.5 (26.43,32.85)	27.3 (24.45,30.42)	5,338	27.24 (26.51,28)	2001–2011	4.97* (2.74,7.26)	< 0.001	2011–2019	−1.32 (−3.69,1.1)	0.259	2.13* (0.63,3.64)	0.005
District of Columbia	9.19 (6.89,12.01)	9.81 (7.54,12.56)	8.95 (6.83,11.53)	1,099	9.1 (8.56,9.67)	2001–2019	0.75 (−0.35,1.86)	0.172				0.75 (−0.35,1.86)	0.172
Florida	19.13 (18.51,19.76)	25.69 (25.08,26.32)	24.48 (23.91,25.07)	104,064	22.58 (22.44,22.72)	2001–2010	1.29* (0.15,2.45)	0.03	2010–2015	4.83* (1.43,8.35)	0.009	1.6* (0.45,2.75)	0.006
						2015–2019	−1.64 (−4.58,1.39)	0.255					
Georgia	18.63 (17.66,19.64)	26.13 (25.16,27.13)	25.36 (24.44,26.31)	42,932	23.97 (23.74,24.2)	2001–2015	2.73* (2.15,3.31)	< 0.001	2015–2019	−2.09 (−5.14,1.06)	0.175	1.64* (0.87,2.41)	< 0.001
Missouri	12.36 (11.47,13.3)	18.95 (17.91,20.03)	17.48 (16.51,18.5)	22,066	17.69 (17.46,17.93)	2001–2005	10.6* (5.13,16.35)	0.001	2005–2017	1.02* (0.11,1.94)	0.032	2.14* (0.38,3.94)	0.017
						2017–2019	−6.88 (−18.55,6.47)	0.266					
Nebraska	15.28 (13.5,17.24)	29.36 (26.99,31.88)	29.9 (27.56,32.39)	7,991	21.72 (21.24,22.21)	2001–2012	1.7* (0.74,2.67)	0.002	2012–2016	10.61* (4.41,17.18)	0.003	3.53* (2.02,5.06)	< 0.001
						2016–2019	1.22 (−3.87,6.58)	0.616					
New Hampshire	23.79 (21.18,26.64)	31.42 (28.73,34.31)	31.01 (28.43,33.78)	8,578	29.89 (29.25,30.55)	2001–2019	0.74 (−0.14,1.62)	0.095				0.74 (−0.14,1.62)	0.095
New Jersey	18.48 (17.59,19.4)	21.27 (20.38,22.19)	21.62 (20.74,22.53)	40,256	21.6 (21.39,21.82)	2001–2006	3.06* (0.4,5.78)	0.027	2006–2019	−0.03 (−0.59,0.53)	0.897	0.81* (0.05,1.58)	0.036
New Mexico	19.62 (17.61,21.79)	16.42 (14.84,18.14)	16.71 (15.13,18.41)	7,275	17.42 (17.02,17.83)	2001–2019	−0.85 (−1.84,0.15)	0.09				−0.85 (−1.84,0.15)	0.09
New York	12.7 (12.2,13.21)	17.99 (17.44,18.56)	18.46 (17.91,19.03)	69,265	16.97 (16.84,17.1)	2001–2006	7.53* (3.66,11.55)	0.001	2006–2019	0.59 (−0.16,1.34)	0.113	2.47* (1.4,3.55)	< 0.001
Oklahoma	12.98 (11.82,14.23)	24.01 (22.54,25.54)	20 (18.69,21.38)	14,267	18.77 (18.46,19.09)	2001–2007	6.23* (2.2,10.42)	0.007	2007–2010	−9.26 (−26.63,12.21)	0.322	1.77 (−1.89,5.56)	0.349
						2010–2016	8.3* (3.66,13.15)	0.003	2016–2019	−7.51 (−15.48,1.21)	0.081		
Rhode Island	22.72 (20.02,25.69)	23.2 (20.55,26.12)	21.56 (19.05,24.32)	5,358	23.14 (22.51,23.78)	2001–2019	−0.12 (−0.89,0.65)	0.741				−0.12 (−0.89,0.65)	0.741
South Carolina	16.27 (15.05,17.56)	24.52 (23.23,25.85)	20.06 (18.95,21.23)	21,829	22.44 (22.14,22.74)	2001–2005	7.5* (0.52,14.97)	0.037	2005–2019	−0.13 (−0.94,0.69)	0.74	1.52* (0.03,3.03)	0.046
North Dakota	12.09 (9.57,15.07)	22.63 (19.35,26.29)	21.11 (18,24.6)	2,810	19.87 (19.13,20.64)	2001–2010	8.63 (5.88,11.46)	< 0.001	2010–2019	−0.04 (−2.02,1.98)	0.967	4.21* (2.67,5.77)	< 0.001
Tennessee	11.4 (10.55,12.3)	19.57 (18.58,20.61)	22.37 (21.33,23.45)	25,682	19.5 (19.26,19.74)	2001–2005	19.9* (7.94,33.18)	0.002	2005–2019	0 (−1.19,1.2)	1	4.11* (1.75,6.53)	0.001
Texas	14.65 (14.11,15.21)	13.44 (13,13.89)	15.79 (15.34,16.26)	62,683	13.99 (13.88,14.11)	2001–2016	−0.86* (−1.5,−0.22)	0.012	2016–2019	5.96 (−0.75,13.11)	0.079	0.24 (−0.86,1.36)	0.668
Utah	21.67 (19.52,23.98)	41.57 (39.12,44.13)	43.43 (41.02,45.94)	14,931	34.5 (33.94,35.06)	2001–2014	4.79* (3.97,5.61)	< 0.001	2014–2019	0.62 (−1.75,3.06)	0.586	3.61* (2.79,4.44)	< 0.001
Vermont	22.55 (19.02,26.57)	41.81 (37.23,46.83)	32.93 (28.83,37.48)	4,537	32.48 (31.52,33.47)	2001–2003	22.64 (−8.29,64)	0.144	2003–2008	−4.01 (−11.52,4.13)	0.279	2.17 (−1.96,6.48)	0.308
						2008–2017	4.59* (2.01,7.24)	0.003	2017–2019	−10.46 (−28.79,12.6)	0.299		
Wisconsin	15.47 (14.45,16.55)	25.43 (24.2,26.7)	23.19 (22.03,24.39)	24,674	20.7 (20.44,20.97)	2001–2003	−3.25 (−16.73,12.41)	0.625	2003–2010	5.99* (3.56,8.47)	< 0.001	2.12* (0.16,4.11)	0.034
						2010–2016	2.47 (−0.13,5.14)	0.06	2016–2019	−3.62 (−8.82,1.88)	0.164		
3. Decreasing trends in recent years													
Connecticut	23.74 (22.18,25.38)	20.79 (19.43,22.23)	19.03 (17.77,20.37)	17,080	21.96 (21.62,22.29)	2001–2005	3.48 (−0.94,8.09)	0.115	2005–2019	−2* (−2.62,−1.38)	< 0.001	−0.81 (−1.79,0.18)	0.109
Indiana	17.1 (16.08,18.17)	24.32 (23.18,25.5)	17.97 (17.02,18.96)	25,226	19.32 (19.08,19.56)	2001–2013	0.91* (0.14,1.7)	0.025	2013–2016	9.96 (−1.68,22.98)	0.089	0.51 (−1.41,2.46)	0.606
						2016–2019	−9.61* (−14.64,−4.29)	0.003					
Massachusetts	21.46 (20.36,22.6)	25.03 (23.93,26.18)	15.09 (14.25,15.96)	30,853	21.83 (21.58,22.08)	2001–2006	3.02* (0.08,6.04)	0.046	2006–2014	−2.67* (−4.3,−1)	0.006	−2.26 (−4.52,0.05)	0.056
						2014–2017	10.21 (−2.11,24.08)	0.095	2017–2019	−27.2* (−36.77,−16.19)	0.001		
Oregon	24.2(22.6,25.87)	25.7(24.25,27.22)	25.1(23.72,26.54)	21,564	26.27 (25.91,26.62)	2001–2014	1.58* (0.59,2.59)	0.004	2014–2019	−4.03* (−7.6,−0.32)	0.036	−0.01 (−1.16,1.16)	0.991
Pennsylvania	16.6 (15.91,17.3)	23.69 (22.91,24.49)	20.06 (19.35,20.79)	58,644	20.68 (20.51,20.86)	2001–2008	1.17 (−0.34,2.71)	0.118	2008–2014	5.98* (3.72,8.3)	< 0.001	0.93 (−0.05,1.92)	0.063
						2014–2019	−5.13* (−7.11,−3.12)	< 0.001					

*PY* Person-years, *APC* Average annual percent changes, *AAPC* average *APC* *CI* Confidence interval, *CM* Cutaneous Melanoma

^a^No available incidence data in 2001, and the values are presented based on the incidence in 2003

^b^No available incidence data in 2018 and 2019. In Table 1, incidence rates are shown for 2001, 2016, and 2019 because these represent the first comparable year under ICD-O-3 coding, the national joinpoint year, and the last pre-pandemic year of the study period, respectively

*Joint point software selected joint point with significant annual percentage changes (*P* < 0.05)

(Table 1). In 2001, the highest CM incidence rate was observed in Oregon (24.2 per 100 K PY, 95% CI 22.6–25.87) and the lowest in the District of Columbia (9.19 per 100 K PY, 95% CI 6.89–12.01), yielding an incidence rate ratio (IRR) of 2.63 (95% CI 2.00–3.47) between these two states. In 2016, the highest CM incidence rate was in Vermont (41.81 per 100 K PY, 95% CI 37.23–46.83) and the lowest again in the District of Columbia (9.81 per 100 K PY, 95% CI 7.54–12.56), with an IRR of 4.26(95% CI 3.25–5.58). In 2019, the highest CM incidence rate was in Utah (43.43 per 100 K PY, 95% CI 41.02–45.94) and the lowest in the District of Columbia (8.95 per 100 K PY, 95% CI 6.83–11.53), with a further increase in the IRR to 4.85(95% CI 3.74–6.29) (Table 1).

Among the 50 states with evaluable recent mortality trends, 13 showed no significant change and 37 showed significant decreases, while no state showed a significant increase. Mortality trend estimates for the District of Columbia were unavailable because of suppressed small-count data. The MRR between the highest and lowest mortality rates by state in 2001,2013, 2017, and 2019 was 2.53 (95% CI 1.49–4.27), 2.29(95% CI 1.47–3.58), 3.71(95% CI 1.97–6.94), and 1.19 (95% CI 0.70–2.02), respectively (Supplementary Table 2).

### National population-level disparities in cutaneous melanoma incidence and mortality by age, sex, and race/ethnicity

**Fig. 1:**
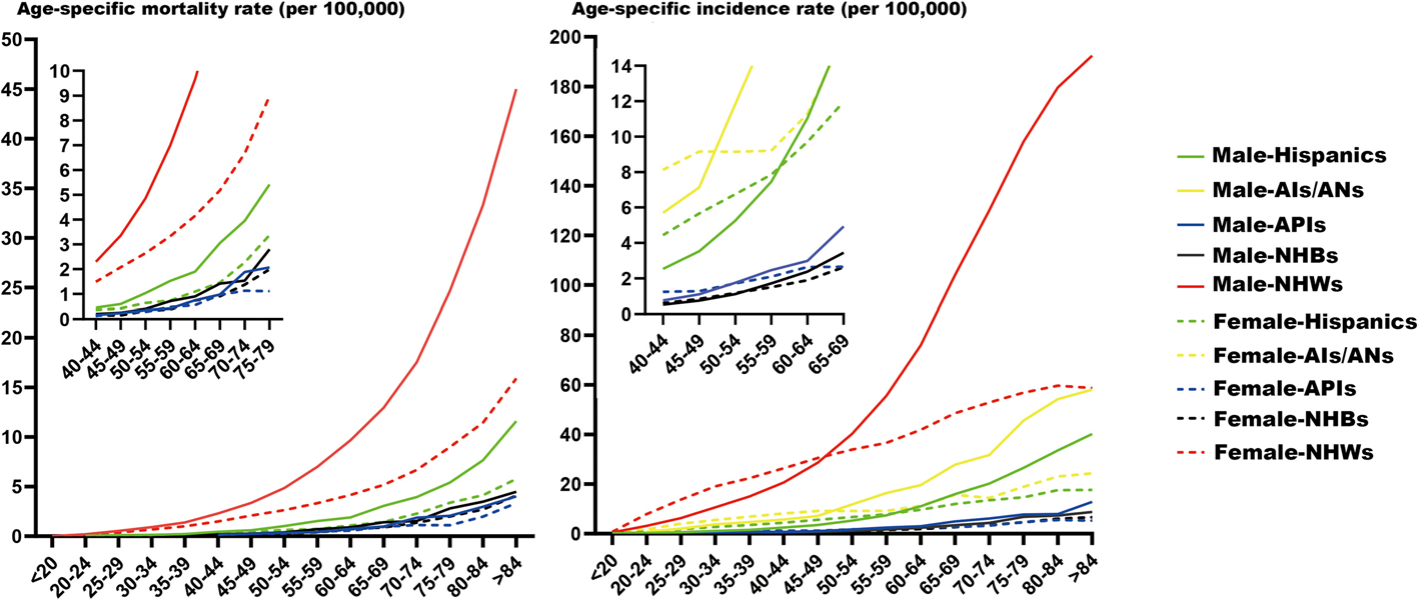
Age-specific cutaneous melanoma (CM) incidence and mortality rates (per 100,000) by sex and race/ethnicity, United States, 2001–2019.Rates are shown for 5-year age groups (< 20, 20–24, …, > 84 years).NHWs, non-Hispanic Whites; NHBs, non-Hispanic Blacks; APIs, non-Hispanic Asians/Pacific Islanders; AIs/ANs, non-Hispanic American Indians/Alaska Natives

Across all racial/ethnic groups, CM incidence in younger adults was higher in females than in males. Among NHWs, non-Hispanic Asian/Pacific Islanders (APIs), and non-Hispanic American Indians/Alaska Natives (AIs/ANs), male incidence rates exceeded female beginning at ages 50–54 years; followed by non-Hispanic Blacks (NHBs), this crossover occurred at ages 55–59 years, and in Hispanics at ages 60–64 years. Among Hispanics, male CM mortality rates surpassed female rates starting at ages 25–29 years. Among NHWs, male CM mortality rates were consistently higher than female across their entire lifespan (Fig. 1).

**Fig. 2:**
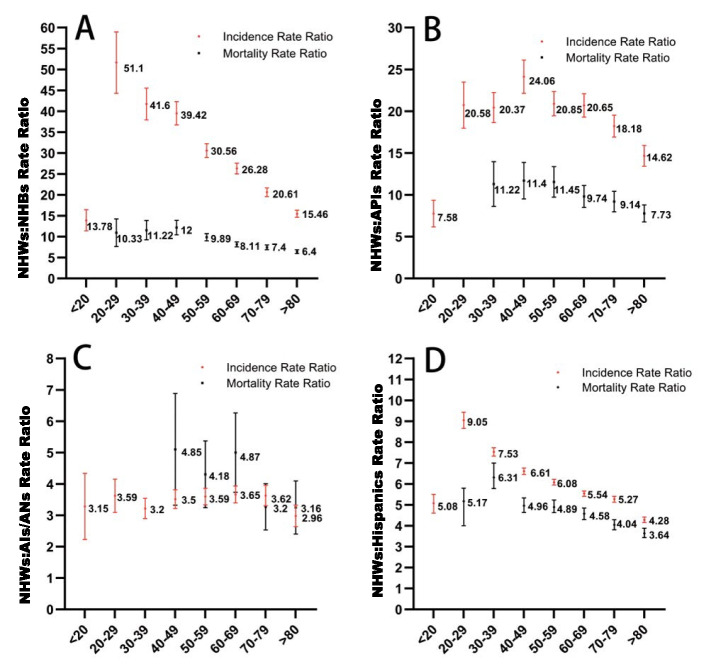
Rate Ratios Comparing CM Incidence Rates and Mortality Rates (2001–2019) in Non-Hispanic Whites and other races/ethnicities by Age. Other races/ethnicities served as the reference group. Error bars indicate 95% confidence intervals. **A**: The Incidence and Mortality Rate Ratio between Non-Hispanic Whites and Non-Hispanic Blacks; **B**: The Incidence and Mortality Rate Ratio between Non-Hispanic Whites and Non-Hispanic Asians/Pacific Islanders; **C**: The Incidence and Mortality Rate Ratio between Non-Hispanic Whites and Non-Hispanic American Indians/Alaska Natives; **D**: The Incidence and Mortality Rate Ratio between Non-Hispanic Whites and Hispanics. NHWs, Non-Hispanic Whites. NHBs, Non-Hispanic Blacks. APIs, Non-Hispanic Asians/Pacific Islanders. AIANs, Non-Hispanic American Indians/Alaska Natives

Figure 2 shows age-specific rate ratios comparing CM Incidence and mortality between NHWs and non-NHWs. Young adults aged 20–29 years had the most significant differences in the CM incidence were observed for NHWs/NHBs and NHWs/Hispanics, and these differences gradually decreased at older ages. For NHWs/APIs, the greatest disparity in incidence was seen at ages 40–49 years. The differences in CM incidence between NHWs and AIs/ANs were small across all age groups. Across racial/ethnic groups, IRRs were smallest in individuals younger than 20 years and in those aged 80 years and older. A similar pattern was observed for mortality, with the smallest MRR in adults aged 80 years and older. Overall, IRRs and MRRs comparing NHWs with non-NHWs decreased with increasing age (Fig. 2).

### State-level variation in racial/ethnic incidence rate ratios

**Fig. 3:**
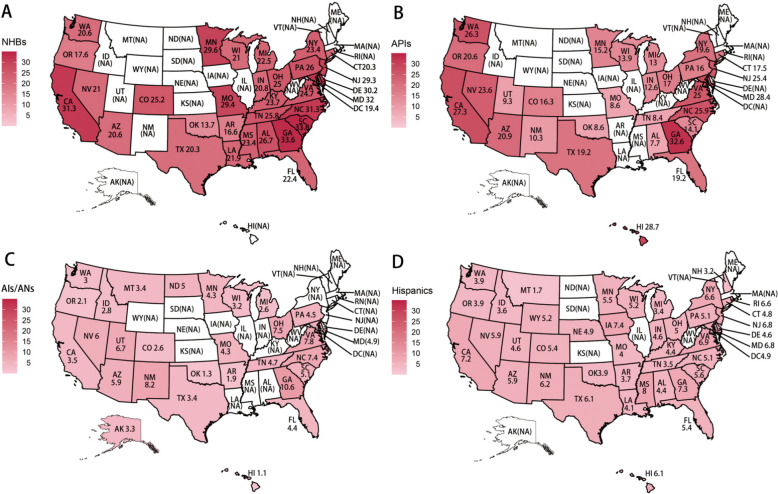
State-level incidence rate ratio (IRR) between Non-Hispanic Whites and other races/ethnicities during 2001–2019. **A**: Incidence Rate Ratio between Non-Hispanic Whites and Non-Hispanic Blacks during 2001–2019; **B**: Incidence Rate Ratio between Non-Hispanic Whites and Non-Hispanic Asians and Pacific Islanders during 2001–2019; **C**: Incidence Rate Ratio between Non-Hispanic Whites and Non-Hispanic American Indian/Alaska Natives during 2001–2019; **D**: Incidence Rate Ratio between Non-Hispanic Whites and Hispanics during 2001–2019. NA Indicates the IRRs in the states are not available

Figure 3 shows state-level IRRs comparing CM incidence in NHWs and non-NHWs over the study period. The highest IRRs for NHWs/NHBs were observed in South Carolina (IRR 33.8, 95% CI 29.3–39.1), followed by Georgia (IRR 33.6, 95% CI 30.4–37.1) and Maryland (IRR 32.0, 95% CI 28.4–36.0). Whereas the lowest IRR was in Oklahoma (IRR 13.7, 95% CI 10.6–17.8) (Fig. 3A). The highest IRR for NHWs/APIs was in Georgia (IRR 32.6, 95% CI 24.3–43.6), followed by Hawaii (IRR 28.7, 95% CI 26.1–31.5) and Maryland (IRR 28.4, 95% CI 22.2–36.3). The smallest IRR was in Alabama (IRR 7.7, 95% CI 4.9–12.3) (Fig. 3B). For NHWs versus AIs/ANs, the highest IRRs were in Georgia (IRR 10.6, 95% CI 6.5–17.2), New Mexico (IRR 8.2, 95% CI 6.6–10.1), Virginia (IRR 7.8, 95% CI 4.8–12.7), whereas the lowest was in Hawaii (IRR 1.1, 95% CI 0.78–1.56) (Fig. 3C). For NHWs/Hispanics, the highest IRRs were in Mississippi (IRR 8.0, 95% CI 5.2–12.3),, Iowa (IRR 7.4, 95% CI 5.4–10.2), and Georgia (IRR 7.3, 95% CI 6.3–8.4), and the lowest was in Montana (IRR 1.7, 95% CI 1.3–2.3) (Fig. 3D).

### State-level ecological analyses of obesity, physical activity, and CM incidence trends

**Fig. 4:**
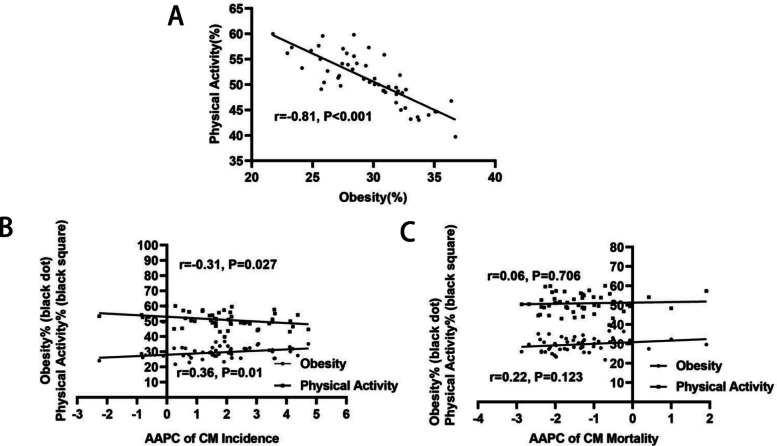
Association between state-level obesity, physical activity and average annual percent changes (AAPC) of CM incidence/mortality rates during 2001–2019. **A**: The association between average of obesity and physical activity percentage. X axis denotes the average of obesity. Y axis represents average percentage of the physical activity. **B**: The association between average of obesity, physical activity percentage and AAPC of CM incidence. X axis denotes AAPC of MOTS incidence. Y axis represents average percentage of obesity and physical activity. **C**: The association between average of obesity, physical activity percentage and AAPC of MOTS mortality. X axis denotes AAPC of MOTS mortality. Y axis represents average percentage of obesity and physical activity. The Y axis of the Black dot denotes average percentage of state-level obesity during 2011–2019, the Y axis of the Black square denotes average percentage of state-level physical activity during 2011–2019. Each Black dot and Black square denotes states and the District of Columbia

Using states (and the District of Columbia) as the unit of analysis, we observed a strong inverse correlation between state-level physical activity and obesity prevalence (*r* = −0.81, *p* < 0.001) (Fig. 4A). In addition, a weak correlation was shown between state-level obesity and the AAPC of CM incidence (*r* = 0.36, *P* = 0.01), physical activity also had a weak inverse correlation with the state-level AAPC of CM incidence (*r* = −0.31, *P* = 0.027) (Fig. 4B). Physical activity, obesity, and the AAPC of CM mortality do not have an ecological correlation (Fig. 4C).

Subgroup analysis showed a weak positive correlation between state-level male obesity and AAPC in CM incidence (*r* = 0.34, *P* = 0.015), and a weak inverse association between male physical activity level and AAPC (*r* = −0.38, *P* = 0.008) (Supplementary Fig. 5 A). Among NHWs, obesity prevalence showed a moderate positive correlation with AAPC in CM incidence (*r* = 0.4, *P* = 0.005), and physical activity levels showed a moderate inverse correlation (*r* = −0.47, *P* = 0.001) (Supplementary Fig. 5E). No meaningful associations were observed in other racial/ethnic subgroups or among females (Supplementary Fig. 5).

Because state-level obesity prevalence and physical activity were strongly inversely correlated (*r* = − 0.81; *p* < 0.001), we fitted two parallel multivariable ecological linear regression models with state-level AAPC in CM incidence as the dependent variable, alternately treating obesity prevalence and physical activity as the primary lifestyle exposure. In the model including obesity prevalence (Supplementary Table 3), higher obesity prevalence was associated with a higher AAPC (β = 0.198, 95% CI 0.059–0.336; *p* = 0.006), whereas a higher personal doctor rate was associated with a lower AAPC (β = − 14.685, 95% CI 26.163—3.208; *p* = 0.013). Health care coverage (β = 19.006, 95% CI 0.322—38.333; *p* = 0.054) and the proportion of non-Hispanic Whites (β = 0.035, 95% CI: 0.004—0.073; *p* = 0.074) showed borderline positive associations. Model fit was modest (R^2^ = 0.324; adjusted R^2^ = 0.209); variance inflation factors ranged from 1.37 to 4.29, and Moran’s I for the residuals (I = 0.07; permutation *p* = 0.51) did not indicate spatial autocorrelation.

In the alternative model with physical activity in place of obesity (Supplementary Table 4), higher physical activity was associated with a lower AAPC (β = − 0.156, 95% CI: 0.246 − 0.066; *p* = 0.001). In this model, higher health care coverage (β = 19.090, 95% CI 1.139–37.040; *p* = 0.038) and a higher proportion of non-Hispanic Whites (β = 0.053, 95% CI 0.016–0.089; *p* = 0.006) were positively associated with AAPC, and the personal doctor rate again showed an inverse association (β = − 14.911, 95% CI 25.826 − 3.996; *p* = 0.009). Average daily solar insolation, indoor tanning restrictions for minors, and school sunscreen policies were not significantly associated with AAPC. Model fit was slightly improved (R^2^ = 0.375; adjusted R^2^ = 0.269); variance inflation factors ranged from 1.33 to 4.34, and Moran’s I for the residuals (I = 0.035; permutation *p* = 0.72) again did not indicate spatial autocorrelation, so additional spatial regression models were not undertaken.

## Discussion

In this nationwide study, which combined state-level geographic analyses with national population-level analyses, the contemporary burden of CM in the US varied markedly across states and population groups. Overall, CM incidence in the US has reached a plateau. Mortality has declined substantially. However, state-level incidence trend varied substantially, with approximately half of the states showing an upward trend. The disparity in incidence between the states with the highest and lowest rates continues to widen. In addition, CM burden varied markedly across subgroups defined by age, sex, and race/ethnicity. These findings imply that the observed geographic and population disparities likely reflect the combined influence of behavioral risk factors, primary prevention environments, and the distribution of healthcare resources across states.

At the national level, CM incidence rates in the US, as well as the subgroups categorized by age, sex, and races/ethnicities, now appears to be leveling off. However, state-level incidence trends remain highly heterogeneous, and recent segmented (joinpoint) analyses indicate that nearly half of states still have rising incidence. In addition, disparities in state-level incidence rates have widened over time, with the IRR between states with the highest and lowest CM incidence increasing across 2001, 2016, and 2019. CM is a disease characterized by prominent geographic variations [4]. Exposure to UV radiation is widely regarded as the most important environmental risk factor for melanoma [29]. CM incidence is closely related to latitude [5]. In the southern US, closer proximity to the equator generally results in higher ambient UV levels, and outdoor recreation and sun-seeking vacation patterns may further increase UV exposure and sunburn risk. Recent work suggests that the potential UV dose received by the average person in the continental US has increased over time [30]. The comparisons of mean annual regional summer UV from 2003 to 2019 using ANOVA with post-hoc TukeyKramer test have shown that southern regions have significantly higher mean UV levels than other regions [30]. In parallel, indoor tanning has been particularly prevalent in the Midwest, where national survey data show the highest regional rates of use among US adults [31]. Indoor tanning may therefore have an important impact on melanoma occurrence in the Midwest region. One study reported that women living in the Midwest and South were more likely to report indoor tanning [32]. The Midwest has also been shown to have the highest per capita density of indoor tanning facilities in the US [32, 33]. Another study suggested that indoor tanning may contribute disproportionately to melanoma risk in the Midwest, where adult indoor tanning prevalence in 2005 (13.1%) was higher than in the South (7.2%), Northeast (6.8%) and West (6.6%) [7]. In Our study, as of 2019, 8 of the 12 Midwestern states still lacked laws allowing students to carry and self-apply sunscreen in schools (supplementary Fig. 4). Furthermore, only 6 states nationwide had no legal restrictions on indoor tanning for minors in 2019, with 4 of which were in the Midwest (supplementary Fig. 2). However, state-level legislation has been shown to substantially reduce CM incidence among young people in the US [34]. Taken together, these geographic differences in UV exposure, indoor tanning patterns, and prevention-related policies may help shape state-level trends in CM incidence.

Our ecological correlation analysis results suggest that higher obesity and lower physical activity level were associated with higher CM incidence. Taken together, these patterns are consistent with previous reports suggesting that excess energy balance may be related to CM risk [35]. In multivariable models adjusted for covariates, higher state-level obesity prevalence and lower physical activity were each significantly associated with a higher AAPC in CM incidence. These findings suggest that energy balance–related metabolic factors may play a role in the observed increases in CM incidence. However, given the ecological study design and reliance on state-level aggregated data, exposure–outcome relationships at the individual level cannot be inferred. In our models, higher personal doctor rates were significantly associated with lower state-level AAPCs, whereas higher health care coverage was only marginally associated with increasing AAPCs. This pattern suggests that higher personal doctor rates may serve as a marker of a more mature primary care system, which facilitates counseling on sun-protective behaviors and prompt evaluation of suspicious skin lesions, and may thereby help dampen the upward trend in CM incidence over time. By contrast, higher health care coverage reflects broader insurance enrollment and improved access to dermatologic assessment. In some states, the expansion of health care coverage after 2010 likely increased the detection of early-stage CM and contributed to steeper rises in reported incidence [36]. In our models, policy-related variables were not statistically significant. One plausible explanation is that most tanning-related legislation was implemented after 2009 [37], when indoor tanning was already widespread and CM incidence was increasing most rapidly. In addition, many of these policies are relatively modest in scope, and their real-world impact likely depends on the strength of enforcement and local implementation, resulting in considerable variability in effectiveness. Overall, the geographic overlap of high obesity prevalence and higher CM incidence in the Midwest and South [38], together with our multivariable ecological findings, supports a potential role for energy-balance-related factors in CM trends, although these ecological associations are subject to residual confounding and do not establish causality.

Our study indicates that CM mortality in the US has been declining steadily at both the national and state levels, accompanied by a marked narrowing of interstate differences in mortality. This pattern aligns closely with the profound shift in melanoma treatment over the past decade. The widespread adoption of immune checkpoint inhibitors and BRAF/MEK–targeted therapies has substantially improved long-term survival among patients with metastatic and advanced CM, and multiple national analyses have documented an accelerated decline in melanoma mortality since the mid-2010s [2, 39]. In contrast to incidence, which is driven primarily by UV exposure and behavioral patterns, mortality more directly reflects variation in access to and quality of treatment. Harmonized clinical practice guidelines and insurance coverage policies have helped facilitated the dissemination of key systemic therapies and standardized treatment pathways across most states. Together, these changes may have helped reduced the overall mortality burden and helped narrowed the gap between high- and low-mortality states. Our findings suggest that, in the era of immunotherapy and targeted therapy, the US has achieved gains in reducing CM-related mortality and may have made progress toward narrowing geographic inequities in melanoma survival. Overall, our findings indicate that state-level CM incidence is becoming more heterogeneous, whereas mortality is becoming more homogeneous, underscoring the need for state-specific prevention strategies alongside equitable access to effective therapies.

Our analyses further describe how the burden of CM is distributed across population subgroups defined by age, sex, and race/ethnicity. Consistent with previous research, our findings indicate that, across all racial and ethnic groups, CM incidence is higher in young women than in young men [20]. This pattern is likely attributable to young women’s greater engagement in intentional outdoor tanning [40], more frequent use of ultraviolet tanning beds and sunbathing [37], hormone-related physiological differences [41], and more proactive skin self-examination [42]. In addition, we systematically quantified, for each racial and ethnic group, the age at which CM incidence in men surpasses that in women. We found that the age at which male CM incidence exceeds female incidence was latest in Hispanics, intermediate in NHBs, and earlier in NHWs, APIs, and AIs/ANs. Wu et al. observed that the crossover age for CM incidence between NHWs and NHBs was later than that among Hispanics. Nevertheless, their study was based on data from the 1999–2006 period, and it has been quite a long time since then. Hence, the conflicting results are not unexpected [6].

Several social and behavioral factors may help explain this delayed crossover. In the US, Hispanics and NHBs populations generally experience economic disadvantages, including lower incomes and reduced access to healthcare, which can lead to female patients detecting tumors at a later stage compared to those from more economically well-off racial/ethnic groups. US cultural norms favor sunscreen use than Hispanic cultural norms [43, 44]. Acculturation among Hispanics has been associated with higher perceived benefits of exposure to UV radiation, less worry about skin damage [45], higher prevalence of indoor tanning and sunbathing [46], and a higher sunburn risk [47, 48]. Obesity is more prevalent among Hispanics and NHBs populations in the US, and female obesity rates are substantially higher than in males [49]. This delayed crossover is more plausibly explained by the combined effects of cultural patterns of sun exposure and photoprotection, the burden of obesity among women, and long-term disadvantages in income and access to dermatologic care, rather than with purely biological differences alone. At the same time, we found that in nearly all racial and ethnic groups, CM mortality in young men consistently exceeds that in young women. This pattern has been attributed by some investigators to men’s lower frequency of skin self-examination [42], as well as sex-related differences in sex hormones, immune homeostasis, vitamin D metabolism, and oxidative stress [22]. Taken together, these results suggest that the life-course patterns of CM burden differ across racial and ethnic groups and highlights the need to tailor prevention and early detection strategies to specific age groups and population characteristics.

In addition, our age-stratified IRRs and MRRs further show that inequities in CM burden between NHWs and non-NHWs are most pronounced in early and mid-adulthood and gradually attenuate with advancing age. Among individuals aged 20–39 years, NHWs have substantially higher incidence and mortality than most non-NHW groups. This pattern is consistent with the high prevalence of indoor tanning among young White populations in the US and the greater susceptibility of fair skin to UV-induced melanoma. With increasing age, tanning tends to decline [50], whereas cumulative lifetime UV exposure becomes the predominant driver of risk [51]. In parallel, Medicare coverage and more stable pension income progressively narrow racial/ethnic differences in access to diagnosis and treatment among older adults [52]. This life-course pattern is also consistent with our state-level findings. States with the largest NHW/non-NHW IRRs (e.g., Georgia, South Carolina, Mississippi, Iowa, New Mexico, Hawaii, and Virginia) tend to have long-standing high UV levels, relatively permissive or historically absent regulations on indoor tanning among minors, and, until recently, lagging legislation allowing students to carry and self-apply sunscreen at school (Supplementary Figs. 1, 2, 3 and 4). There is a strong alignment between these population-level gradients and state-level geographic and policy characteristics, suggesting that UV exposure environments and gaps in photoprotective policies at the state level may help shape CM inequities. These contextual factors may amplify racial/ethnic inequities in CM burden by influencing sun exposure and tanning behaviors in youth and early adulthood and by affecting opportunities for early detection.

### Limitations

This study has several limitations. First, this was an ecological analysis based on state-level aggregated data; therefore, the observed associations cannot be interpreted at the individual level and are subject to ecological fallacy. Second, although we adjusted for a range of state-level sociodemographic, environmental, and healthcare related covariates, important factors such as individual socioeconomic status, skin phototype, nevus burden, tumor thickness, dermatologist density, and detailed sun-exposure behaviors were unavailable, and residual confounding is likely. Third, key exposures (e.g., obesity, health care coverage, personal doctor rate) were derived from self-reported BRFSS data, and solar insolation and tanning/sunscreen policies were only crude proxies for UV exposure and primary-prevention environments, which may have introduced misclassification and biased associations toward the null. Fourth, because of strong collinearity between obesity and physical activity, these two variables could not be included simultaneously in the same multivariable model. Therefore, obesity and physical activity were evaluated in separate models, and the obesity- or physical activity–AAPC association should be interpreted as reflecting broader aspects of energy-balance environments rather than the isolated effect of either variable alone. Finally, the number of analytic units was limited (50 states plus the District of Columbia), and race/ethnicity was categorized using registry-based social groupings, so some age- and race/ethnicity-specific IRRs and MRRs are imprecise. In addition, our findings may not generalize beyond the US context during 2001–2019.

## Conclusions

In the United States, cutaneous melanoma incidence appears to have plateaued at the national level in recent years, whereas mortality has declined. However, substantial state-level heterogeneity remains, with interstate incidence disparities widening over time even as interstate mortality disparities have narrowed. In ecological analyses, state-level obesity prevalence, physical activity, and personal doctor rates were associated with CM incidence trends, although these associations should be interpreted cautiously and do not establish causality. In addition, CM burden varied markedly across age, sex, and racial/ethnic subgroups, with substantial between-state variation in racial/ethnic incidence rate ratios. Overall, our findings highlight persistent geographic and population-level disparities in CM in the US and provide a more detailed basis for future surveillance, risk stratification, and hypothesis-driven prevention research.

## Supplementary Information

Supplementary Material 1. Supplementary Table 1. Age-adjusted CM incidence/mortality rates and joinpoint trends, 2001-2019, by overall, sex, age, races/ethnicities. PY, person-years; APC, average annual percent changes; AAPC, average APC; CI, confidence interval; CM, Cutaneous Melanoma; NHWs, Non-Hispanic Whites. NHBs, Non-Hispanic Blacks. APIs, Non-Hispanic Asians/Pacific Islanders. AIs/ANs, Non-Hispanic American Indians/Alaska Natives; *Joint point software selected joint point with significant annual percentage changes (*P* < 0.05).

Supplementary Material 2. Supplementary Table 2. Age-adjusted CM mortality rates and joinpoint trends, 2001-2019, by states. PY, person-years; APC, annual percent changes; AAPC, average APC; CI, confidence interval; CM, Cutaneous Melanoma. *Joint point software selected joint point with significant annual percentage changes (*P* < 0.05); ^a^Mortality rate data is available only for the years 2010, 2014, and 2015. ^b^Mortality rate data is available only for the years 2006, 2009, 2011, 2013, 2014, 2015, 2017 and 2019. ^c^Mortality rate records are unavailable for 2001, 2005, and 2012. Mortality trend estimates for the District of Columbia were unavailable because annual death counts were too small and were suppressed in the source database, precluding APC/AAPC estimation.

Supplementary Material 3. Supplementary Table 3. Multivariable linear regression models of state-level AAPC in cutaneous melanoma incidence (2001–2019), with obesity prevalence as the primary exposure. AAPC, average annual percent change in age-adjusted melanoma incidence (2001–2019). β indicates the absolute change in AAPC (percentage points per year) per unit increase in each covariate. Minor indoor tanning policy reflects state-level restrictions on indoor tanning for minors. School sunscreen policy reflects laws allowing students to carry and self-apply sunscreen at school. Non-Hispanic White (%) is derived from the 2010 U.S. Census at the state level. UV=ultraviolet. Analysis includes 49 states (Alaska and Hawaii excluded due to missing UV data). *P < 0.05. Supplementary Table 4. Multivariable linear regression models of state-level AAPC in cutaneous melanoma incidence (2001–2019), with physical activity level as the primary exposure. AAPC, average annual percent change in age-adjusted melanoma incidence (2001–2019). β indicates the absolute change in AAPC (percentage points per year) per unit increase in each covariate. Minor indoor tanning policy reflects state-level restrictions on indoor tanning for minors. School sunscreen policy reflects laws allowing students to carry and self-apply sunscreen at school. Non-Hispanic White (%) is derived from the 2010 U.S. Census at the state level. UV=ultraviolet.Analysis includes 49 states (Alaska and Hawaii excluded due to missing UV data). *P < 0.05.

Supplementary Material 4. Supplementary Figure 1: State-level legislation on indoor tanning restrictions for minors in 2009.

Supplementary Material 5. Supplementary Figure 2: State-level legislation on indoor tanning restrictions for minors in 2019.

Supplementary Material 6. Supplementary Figure 3: State-level laws allowing students to carry and self-apply sunscreen in schools in 2001.

Supplementary Material 7. Supplementary Figure 4: State-level laws allowing students to carry and self-apply sunscreen in schools in 2019. Supplementary Figure 1-4. State-level indoor tanning and school sunscreen policies in selected years. These policy maps were downloaded directly from the CDC's National Environmental Public Health Tracking Network. Minor indoor tanning restrictions and school sunscreen policies were included as covariates in the multivariable ecological models of state-level AAPC in CM incidence (Supplementary Tables 3 and 4). Access the data via the following URL: https://ephtracking.cdc.gov/DataExplorer/?c=19.

Supplementary Material 8. Supplementary Figure 5: Association between state-level obesity, physical activity and average annual percent changes (AAPC) of MOTS incidence/mortality during 2001-2019, by sex and race/ethnicity. A: Obesity, Physical Activity, and AAPC of male incidence; B: Obesity, Physical Activity, and AAPC of female incidence; C: Obesity, Physical Activity, and AAPC of male mortality; D: Obesity, Physical Activity, and AAPC of female mortality; E: Obesity, Physical Activity, and AAPC of Non-Hispanic Whites incidence; F: Obesity, Physical Activity, and AAPC of Non-Hispanic Blacks incidence. G: Obesity, Physical Activity, and AAPC of Hispanic incidence; H: Obesity, Physical Activity, and AAPC of Non-Hispanic Whites mortality. The X axis denotes AAPC of CM incidence or mortality. The Y axis of the Black dot denotes average percentage of state-level obesity during 2011-2019, the Y axis of the Black square denotes average percentage of state-level physical activity during 2011-2019. Each Black dot and Black square denotes states and the District of Columbia.

## Data Availability

All data were publicly obtained from the United States Cancer Statistics, CDC's National Environmental Public Health Tracking Network, and Behavioral Risk Factor Surveillance System. The data can be downloaded from the following 3 websites, respectively: https://wonder.cdc.gov/cancer-archives.HTML https://ephtracking.cdc.gov/DataExplorer/?c=19 https://www.cdc.gov/brfss/brfssprevalence/

## Abbreviations

CM: Cutaneous Melanoma
USCS: US Cancer Statistics
NHWs: Non-Hispanic Whites
MRR: Mortality rate ratio
IRR: Incidence rate ratio
UV: Ultraviolet
NEPHTN: National Environmental Public Health Tracking Network
BRFSS: Behavioral Risk Factor Surveillance System
BMI: Body mass index
APC: Annual percent change
AAPC: Average APC
PY: Person-Years
AIs/ANs: Non-Hispani American Indians and Alaska Natives
APIs: Non-Hispanic Asians and Pacific Islanders
NHBs: Non-Hispanic Blacks
