# Supplementary material for: Geographic and population disparities in cutaneous melanoma in the United States: state-level trends and national population-level analyses
Source: BMC Public Health. 2026 May 2;26:1986. doi: 10.1186/s12889-026-27396-z (PMC13321868; doi:10.1186/s12889-026-27396-z)
Supplement: Supplementary file 1 — Supplementary Material 1. Supplementary Table 1. Age-adjusted CM incidence/mortality rates and joinpoint trends, 2001-2019, by overall, sex, age, races/ethnicities. PY, person-years; APC, average annual percent changes; AAPC, average APC; CI, confidence interval; CM, Cutaneous Melanoma; NHWs, Non-Hispanic Whites. NHBs, Non-Hispanic Blacks. APIs, Non-Hispanic Asians/Pacific Islanders. AIs/ANs, Non-Hispanic American Indians/Alaska Natives; *Joint point software selected joint point with significant annual percentage changes (P < 0.05). [file 12889_2026_27396_MOESM1_ESM.docx]

| **Supplementary Table 1. Age-adjusted CM incidence / mortality rates and joinpoint trends, 2001-2019, by overall, sex, age, races/ethnicities** | | | | | | | | | | | | | | |
| --- | --- | --- | --- | --- | --- | --- | --- | --- | --- | --- | --- | --- | --- | --- |
| Demograp-hic factors | No of new cases | Age adjusted/100 000  (95% CI) | Trend 1 to Trend 4 | | | 2001-2019 |  | No of new deaths | Age adjusted/100 000  (95% CI) | Trend 1 to Trend 4 | | | 2001-2019 |  |
|  | 2001-2019 | 2001-2019 | Years | APC (95%CI) | *P* Value | AAPC  (95%CI) | *P* Value | 2001-2019 | 2001-2019 | Years | APC (95%CI) | *P* Value | AAPC (95%CI) | *P* Value |
| Overall | 1,303,136 | 20.67  (20.64,20.71) | 2001-2016 | 1.95*  (1.67,2.24) | < 0.001 | 1.53*  (1.04,2.01) | < 0.001 | 161,565 | 2.53 (2.52,2.54) | 2001-2013 | 0.15  (-0.21,0.51) | 0.388 | -1.52*  (-2.38,-0.66) | 0.001 |
|  |  |  | 2016-2019 | -0.59  (-3.36,2.27) | 0.662 |  |  |  |  | 2013-2017 | -6.44*  (-9.17,-3.63) | < 0.001 |  |  |
|  |  |  |  |  |  |  |  |  |  | 2017-2019 | -1.38  (-7.27,4.9) | 0.631 |  |  |
| Sex | | | | | | | | | | | | | | |
| Male | 760,085 | 26.47  (26.41,26.53) | 2001-2016 | 2.06*  (1.79,2.32) | <0.001 | 1.51*  (1.06,1.95) | <0.001 | 105,060 | 3.76  (3.73,3.78) | 2001-2009 | 1.02*  (0.47,1.56) | 0.003 | -1.4*  (-2.29,-0.49) | 0.003 |
|  |  |  | 2016-2019 | -1.2  (-3.7,1.35) | 0.327 |  |  |  |  | 2009-2014 | -1.39  (-2.81,0.04) | 0.056 |  |  |
|  |  |  |  |  |  |  |  |  |  | 2014-2017 | -7.5*  (-11.81,-2.98) | 0.005 |  |  |
|  |  |  |  |  |  |  |  |  |  | 2017-2019 | -1.49  (-6.25,3.51) | 0.503 |  |  |
| Female | 543,051 | 16.52  (16.47,16.56) | 2001-2007 | 2.72*  (1.44,4.02) | 0.001 | 1.59*  (0.41,2.78) | 0.008 | 56,505 | 1.6  (1.59,1.62) | 2001-2012 | -0.34  (-0.99,0.32) | 0.286 | -1.84*  (-2.43,-1.25) | <0.001 |
|  |  |  | 2007-2012 | 0.24  (-1.90,2.42) | 0.806 |  |  |  |  | 2012-2019 | -4.16*  (-5.43,-2.88) | <0.001 |  |  |
|  |  |  | 2012-2015 | 3.84  (-2.7,10.82) | 0.218 |  |  |  |  |  |  |  |  |  |
|  |  |  | 2015-2019 | -0.07  (-2,1.9) | 0.938 |  |  |  |  |  |  |  |  |  |
| Age(years) | | | | | | | | | | | | | | |
| <20 | 7,197 | 0.45  (0.44,0.46) | 2001-2019 | -4.08*  (-4.82,-3.33) | <0.001 | -4.08*  (-4.82,-3.33) | <0.001 | - | - | - | - | - | - | - |
| 20-29 | 40,563 | 5.01  (4.96,5.06) | 2001-2007 | 0.72  (-1.22,2.7) | 0.441 | -2.5*  (-3.22,-1.77) | < 0.001 | 1,618 | 0.20  (0.19,0.21) | 2001-2009 | -0.87  (-4.41,2.8) | 0.613 | -5.11*  (-7.16,-3.03) | < 0.001 |
|  |  |  | 2007-2019 | -4.07*  (-4.78,-3.36) | < 0.001 |  |  |  |  | 2009-2019 | -8.38*  (-11.22,-5.44) | < 0.001 |  |  |
| 30-39 | 88,688 | 11.37  (11.30,11.45) | 2001-2019 | -0.18  (-0.5,0.15) | 0.262 | -0.18  (-0.5,0.15) | 0.262 | 5,162 | 0.66  (0.65,0.68) | 2001-2019 | -2.76*  (-3.63,-1.87) | < 0.001 | -2.76*  (-3.63,-1.87) | < 0.001 |
| 40-49 | 157,150 | 19.12  (19.02,19.21) | 2001-2019 | 0.07  (-0.2,0.34) | 0.6 | 0.07  (-0.2,0.34) | 0.6 | 13,379 | 1.62  (1.59,1.65) | 2001-2013 | -2.59*  (-3.25,-1.91) | < 0.001 | -3.87*  (-4.7,-3.03) | < 0.001 |
|  |  |  |  |  |  |  |  |  |  | 2013-2019 | -6.37*  (-8.69,-4) | < 0.001 |  |  |
| 50-59 | 246,719 | 31.87  (31.74,32.00) | 2001-2006 | 2.08*  (0.29,3.91) | 0.028 | 1.1  (-0.1,2.31) | 0.072 | 25,840 | 3.32  (3.28,3.36) | 2001-2012 | -1.6*  (-2.32,-0.87) | < 0.001 | -3.37*  (-4.05,-2.7) | < 0.001 |
|  |  |  | 2006-2012 | 0.22  (-1.35,1.82) | 0.756 |  |  |  |  | 2012-2019 | -6.1*  (-7.58,-4.59) | < 0.001 |  |  |
|  |  |  | 2012-2015 | 3.73  (-2.94,10.86) | 0.240 |  |  |  |  |  |  |  |  |  |
|  |  |  | 2015-2019 | -0.73  (-2.79,1.38) | 0.445 |  |  |  |  |  |  |  |  |  |
| 60-69 | 298,021 | 53.97  (53.77,54.16) | 2001-2016 | 2.55*  (2.24,2.87) | < 0.001 | 2.04*  (1.55,2.53) | < 0.001 | 34,626 | 6.26  (6.19,6.33) | 2001-2010 | 0.61  (-0.3,1.54) | 0.174 | -1.91*  (-2.47,-1.35) | < 0.001 |
|  |  |  | 2016-2019 | -0.47  (-3.19,2.32) | 0.720 |  |  |  |  | 2010-2019 | -4.37*  (-5.18,-3.56) | < 0.001 |  |  |
| 70-79 | 269,446 | 79.32  (79.02,79.62) | 2001-2015 | 3.37*  (3.05,3.68) | < 0.001 | 2.75*  (2.34,3.15) | < 0.001 | 38,546 | 11.37  (11.26,11.48) | 2001-2011 | 1.36*  (0.61,2.11) | 0.002 | -1.4*  (-1.95,-0.85) | < 0.001 |
|  |  |  | 2015-2019 | 0.62  (-1,2.25) | 0.429 |  |  |  |  | 2011-2019 | -4.75*  (-5.7,-3.78) | < 0.001 |  |  |
| ≥80 | 195,352 | 91.95  (91.55,92.36) | 2001-2014 | 4.8*  (4.43,5.17) | < 0.001 | 3.61*  (3.22,4.01) | < 0.001 | 42,217 | 19.7  (19.51,19.89) | 2001-2012 | 2.8*  (2.03,3.58) | < 0.001 | 0.75*  (0.12,1.38) | 0.019 |
|  |  |  | 2014-2019 | 0.58  (-0.6,1.77) | 0.309 |  |  |  |  | 2012-2019 | -2.39*  (-3.66,-1.11) | 0.001 |  |  |
| Race/ethnicity | | | | | | | | | | | | | | |
| AIs/ANs | 3,093 | 7.62  (7.34,7.9) | 2001-2015 | 3.54*  (2.59,4.5) | < 0.001 | 1.98*  (0.77,3.2) | 0.001 | 306 | 0.84  (0.75,0.95) | 2002-2019 | -3.57*  (-5.44,-1.65) | 0.002 | -3.57*  (-5.44,-1.65) | 0.002 |
|  |  |  | 2015-2019 | -3.3  (-7.94,1.57) | 0.165 |  |  |  |  |  |  |  |  |  |
| APIs | 3,881 | 1.37  (1.32,1.41) | 2001-2019 | -0.77*  (-1.37,-0.16) | 0.016 | -0.77*  (-1.37,-0.16) | 0.016 | 891 | 0.34  (0.31,0.36) | 2001-2019 | -1.65*  (-3.04,-0.23) | 0.026 | -1.65*  (-3.04,-0.23) | 0.026 |
| NHBs | 6,403 | 1.03  (1.0,1.06) | 2001-2019 | -0.87*  (-1.47,-0.27) | 0.008 | -0.87*  (-1.47,-0.27) | 0.008 | 2,354 | 0.39  (0.38,0.41) | 2001-2007 | 1.96  (-2.01,6.09) | 0.312 | -1.69*  (-3.11,-0.25) | 0.021 |
|  |  |  |  |  |  |  |  |  |  | 2007-2019 | -3.47*  (-4.75,-2.18) | < 0.001 |  |  |
| NHWs | 1,217,706 | 26.29  (26.24,26.34) | 2001-2016 | 2.36*  (2.05,2.67) | < 0.001 | 1.83*  (1.28,2.37) | < 0.001 | 153,801 | 3.16  (3.14,3.18) | 2001-2013 | 0.56*  (0.19,0.93) | 0.007 | -1.13*  (-2.04,-0.21) | 0.016 |
|  |  |  | 2016-2019 | -0.78  (-3.88,2.41) | 0.604 |  |  |  |  | 2013-2017 | -6.07*  (-8.98,-3.06) | 0.001 |  |  |
|  |  |  |  |  |  |  |  |  |  | 2017-2019 | -1.08  (-7.36,5.62) | 0.722 |  |  |
| Hispanics | 28,352 | 4.6  (4.55,4.66) | 2001-2010 | -0.67  (-1.74,0.42) | 0.204 | -0.19  (-1.21,0.84) | 0.713 | 3,967 | 0.73  (0.71,0.75) | 2001-2019 | -1.18*  (-1.96,-0.39) | 0.006 | -1.18*  (-1.96,-0.39) | 0.006 |
|  |  |  | 2010-2016 | 2.06  (-0.12,4.29) | 0.062 |  |  |  |  |  |  |  |  |  |
|  |  |  | 2016-2019 | -3.18  (-7.27,1.09) | 0.127 |  |  |  |  |  |  |  |  |  |

PY, person-years; APC, average annual percent changes; AAPC, average APC; CI, confidence interval; CM, Cutaneous Melanoma; NHWs, Non-Hispanic Whites. NHBs, Non-Hispanic Blacks. APIs, Non-Hispanic Asians/Pacific Islanders. AIs/ANs, Non-Hispanic American Indians/Alaska Natives; *Joint point software selected joint point with significant annual percentage changes (P < 0.05).
