# Supplementary material for: Geographic and population disparities in cutaneous melanoma in the United States: state-level trends and national population-level analyses
Source: BMC Public Health. 2026 May 2;26:1986. doi: 10.1186/s12889-026-27396-z (PMC13321868; doi:10.1186/s12889-026-27396-z)
Supplement: Supplementary file 2 — Supplementary Material 2. Supplementary Table 2. Age-adjusted CM mortality rates and joinpoint trends, 2001-2019, by states. PY, person-years; APC, annual percent changes; AAPC, average APC; CI, confidence interval; CM, Cutaneous Melanoma. *Joint point software selected joint point with significant annual percentage changes (P < 0.05); aMortality rate data is available only for the years 2010, 2014, and 2015. bMortality rate data is available only for the years 2006, 2009, 2011, 2013, 2014, 2015, 2017 and 2019. cMortality rate records are unavailable for 2001, 2005, and 2012. Mortality trend estimates for the District of Columbia were unavailable because annual death counts were too small and were suppressed in the source database, precluding APC/AAPC estimation. [file 12889_2026_27396_MOESM2_ESM.docx]

|  |  | | | | | | | | | | | | | | |
| --- | --- | --- | --- | --- | --- | --- | --- | --- | --- | --- | --- | --- | --- | --- | --- |
| Supplementary Table 2. Age-adjusted CM mortality rates and joinpoint trends, 2001-2019, by states | | | | | | | | | | | | | | | |
|  | | Mortality rates per 100000 PY | | | | No of death cases | Mortality rates per 100000 PY (95%CI) | Trend1^a^/Trend3 | | | Trend2/Trend4 | | | 2001-2019 |  |
| States | | 2001 | 2013 | 2017 | 2019 | 2001-2019 | 2001-2019 | Years | APC  (95%CI) | P values | Years | APC (95%CI) | P value | AAPC | P value |
| 1.No significant change in recent years | | | | | | | | | | | | | | | |
| Alaska^a^ | |  |  |  |  | 216 | 2.05(1.76,1.76) | 2010-2015 | 1.88  (-51.26,112.98) | 0.802 |  |  |  | 1.88  (-51.26,112.98) | 0.802 |
| Iowa | | 2.18(1.7,2.16) | 2.81(2.28,3.42) | 2.66(2.16,3.24) | 2.73(2.23,3.33) | 1803 | 2.63(2.51,2.51) | 2001-2009 | 4.17*  (1.05,7.38) | 0.012 | 2009-2019 | -1.47  (-3.38,0.48) | 0.127 | 1  (-0.59,2.61) | 0.219 |
| Minnesota | | 2.47(2.05,2.95) | 2.72(2.31,3.18) | 1.82(1.50,2.18) | 2.33(1.98,2.73) | 2586 | 2.33(2.24,2.24) | 2001-2014 | 1.21*  (0.01,2.43) | 0.049 | 2014-2017 | -11.44  (-29.5,11.24) | 0.266 | 0.44  (-3.54,4.59) | 0.831 |
|  | |  |  |  |  |  |  | 2017-2019 | 15.44  (-6.49,42.52) | 0.162 |  |  |  |  |  |
| Mississippi | | 2.23(1.71,2.86) | 2.03(1.57,2.60) | 1.94(1.50,2.48) | 1.91(1.49,2.43) | 1314 | 2.23(2.11,2.11) | 2001-2019 | -0.6  (-1.54,0.36) | 0.204 |  |  |  | -0.6  (-1.54,0.36) | 0.204 |
| Montana | | 3.54(2.47,4.94) | 2.48(1.69,3.53) | 2.52(1.72,3.58) | 2.17(1.46,3.14) | 633 | 2.81(2.59,2.59) | 2001-2019 | -1.12  (-2.48,0.26) | 0.103 |  |  |  | -1.12  (-2.48,0.26) | 0.103 |
| Nebraska | | 3.02(2.26,3.96) | 3.41(2.66,4.31) | 2.21(1.62,2.95) | 2.39(1.79,3.14) | 1087 | 2.82(2.65,2.65) | 2001-2019 | -0.88  (-2.43,0.68) | 0.249 |  |  |  | -0.88  (-2.43,0.68) | 0.249 |
| New Hampshire | | 2.04(1.33,2.99) | 2.73(1.95,3.74) | 3.36(2.49,4.45) | 2.35(1.70,3.20) | 823 | 2.87(2.67,2.67) | 2001-2019 | -1.34  (-2.88,0.22) | 0.088 |  |  |  | -1.34  (-2.88,0.22) | 0.088 |
| New Mexico | | 1.94(1.35,2.72) | 2.43(1.84,3.16) | 2.01(1.48,2.68) | 1.94(1.43,2.59) | 1027 | 2.47(2.32,2.32) | 2001-2019 | -1  (-2.58,0.6) | 0.203 |  |  |  | -1  (-2.58,0.6) | 0.203 |
| North Dakota^b^ | |  | 2.63(1.67,3.98) | 2.43(1.47,3.78) | 1.76(0.98,2.95) | 298 | 1.99(1.76,1.76) | 2006-2019 | -1.53  (-4.22,1.23) | 0.221 |  |  |  | -1.53  (-4.22,1.23) | 0.221 |
| South Dakota | | 3.05(1.96,4.52) | 2.11(1.29,3.28) | 2.46(1.58,3.68) | 1.55(0.89,2.52) | 444 | 2.48(2.25,2.25) | 2001-2019 | -0.98  (-3.38,1.48) | 0.408 |  |  |  | -0.98  (-3.38,1.48) | 0.408 |
| West Virginia | | 2.50(1.87,3.29) | 3.61(2.86,4.51) | 2.89(2.24,3.70) | 2.52(1.91,3.28) | 1334 | 3.1(2.93,2.93) | 2001-2019 | -0.24  (-1.49,1.04) | 0.698 |  |  |  | -0.24  (-1.49,1.04) | 0.698 |
| Wisconsin | | 2.65(2.24,3.12) | 2.37(2.01,2.78) | 2.11(1.78,2.49) | 2.21(1.89,2.58) | 3121 | 2.53(2.44,2.44) | 2001-2019 | -0.6  (-1.44,0.25) | 0.152 |  |  |  | -0.6  (-1.44,0.25) | 0.152 |
| Wyoming^c^ | |  | 2.47(1.39,4.08) | 3.78(2.38,5.69) | 2.30(1.31,3.79) | 347 | 3.07(2.75,2.75) | 2002-2019 | -0.81  (-2.29,0.69) | 0.267 |  |  |  | -0.81  (-2.29,0.69) | 0.267 |
| 2. Decreasing trends in recent years | | | | | | | | | | | | | | | |
| Alabama | | 2.60(2.16,3.11) | 2.68(2.26,3.16) | 2.26(1.89,2.69) | 1.93(1.59,2.33) | 2658 | 2.64(2.54,2.54) | 2001-2009 | 2.16  (-1.16,5.59) | 0.187 | 2009-2019 | -3.96  (-6.22,-1.65) | 0.003 | -1.29  (-3.05,0.51) | 0.16 |
| Arizona | | 2.45(2.04,2.91) | 2.97(2.59,3.39) | 2.19(1.88,2.53) | 2.19(1.90,2.52) | 3630 | 2.73(2.64,2.64) | 2001-2013 | 0.7  (-0.84,2.27) | 0.349 | 2013-2019 | -5.87  (-9.6,-1.98) | 0.006 | -1.54  (-3.05,0) | 0.05 |
| Arkansas | | 2.61(2.05,3.27) | 2.67(2.14,3.29) | 1.58(1.19,2.05) | 1.54(1.18,1.99) | 1528 | 2.45(2.32,2.32) | 2001-2012 | 1.87*  (0.22,3.56) | 0.029 | 2012-2019 | -8.18  (-11.29,-4.96) | 0 | -2.16*  (-3.64,-0.65) | 0.005 |
| California | | 2.56(2.38,2.74) | 2.37(2.21,2.53) | 1.89(1.76,2.03) | 1.85(1.72,1.98) | 16655 | 2.37(2.33,2.33) | 2001-2012 | -0.09  (-0.92,0.74) | 0.812 | 2012-2019 | -5.14  (-6.76,-3.49) | 0 | -2.09*  (-2.84,-1.33) | <0.001 |
| Colorado | | 3.11(2.57,3.73) | 3.22(2.75,3.76) | 2.68(2.28,3.14) | 2.73(2.33,3.18) | 2773 | 2.95(2.84,2.84) | 2001-2009 | 3.33  (-0.71,7.54) | 0.1 | 2009-2019 | -3.85  (-6.36,-1.28) | 0.007 | -0.72  (-2.79,1.39) | 0.499 |
| Connecticut | | 2.94(2.41,3.55) | 2.21(1.78,2.72) | 1.72(1.35,2.16) | 1.83(1.46,2.27) | 1950 | 2.41(2.3,2.3) | 2001-2019 | -2.37*  (-3.27,-1.46) | 0 |  |  |  | -2.37*  (-3.27,-1.46) | <0.001 |
| Delaware | | 3.90(2.66,5.51) | 2.61(1.73,3.80) | 2.40(1.58,3.51) | 2.21(1.45,3.25) | 571 | 2.86(2.63,2.63) | 2001-2019 | -1.3*  (-2.52,-0.06) | 0.041 |  |  |  | -1.3*  (-2.52,-0.06) | 0.041 |
| Florida | | 2.73(2.51,2.98) | 2.95(2.74,3.17) | 2.11(1.94,2.29) | 2.06(1.90,2.24) | 12925 | 2.71(2.67,2.67) | 2001-2012 | 0.66  (-0.33,1.66) | 0.177 | 2012-2019 | -5.73  (-7.49,-3.93) | 0 | -1.88*  (-2.72,-1.02) | <0.001 |
| Georgia | | 2.97(2.59,3.40) | 2.72(2.39,3.08) | 1.87(1.61,2.15) | 1.76(1.52,2.03) | 4065 | 2.36(2.29,2.29) | 2001-2014 | -0.41  (-1.5,0.69) | 0.44 | 2014-2019 | -6.57*  (-10.88,-2.05) | 0.008 | -2.16*  (-3.52,-0.78) | 0.002 |
| Hawaii | | 1.58(0.97,2.45) | 1.64(1.09,2.38) | 1.02(0.59,1.66) | 0.85(0.50,1.38) | 464 | 1.5(1.36,1.36) | 2001-2019 | -1.99*  (-3.75,-0.19) | 0.032 |  |  |  | -1.99*  (-3.75,-0.19) | 0.032 |
| Idaho | | 3.13(2.22,4.27) | 3.75(2.89,4.80) | 2.40(1.75,3.21) | 2.93(2.22,3.80) | 979 | 3.21(3.01,3.01) | 2001-2013 | 0.83  (-1.06,2.74) | 0.365 | 2013-2019 | -5.44*  (-9.98,-0.66) | 0.029 | -1.31  (-3.15,0.58) | 0.173 |
| Illinois | | 2.13(1.88,2.41) | 2.72(2.45,3.01) | 2.04(1.81,2.29) | 1.69(1.49,1.91) | 6013 | 2.33(2.27,2.27) | 2001-2013 | 0.55  (-0.76,1.87) | 0.383 | 2013-2019 | -6.91*  (-10.45,-3.22) | 0.001 | -2*  (-3.39,-0.59) | 0.006 |
| Indiana | | 2.43(2.06,2.86) | 3.21(2.80,3.66) | 2.37(2.03,2.75) | 2.23(1.91,2.60) | 3697 | 2.79(2.7,2.7) | 2001-2010 | 2.2*  (0.03,4.42) | 0.047 | 2010-2019 | -4.1*  (-6.11,-2.04) | 0.001 | -1  (-2.36,0.38) | 0.154 |
| Kansas | | 2.73(2.15,3.42) | 3.40(2.78,4.11) | 2.18(1.71,2.75) | 2.18(1.71,2.73) | 1689 | 2.84(2.7,2.7) | 2001-2013 | 1.53  (-0.41,3.51) | 0.114 | 2013-2019 | -6.62*  (-11.43,-1.54) | 0.015 | -1.26  (-3.21,0.73) | 0.212 |
| Kentucky | | 3.90(3.32,4.56) | 3.39(2.88,3.95) | 2.69(2.26,3.18) | 2.34(1.94,2.80) | 2810 | 3.12(3,3) | 2001-2019 | -1.51*  (-2.46,-0.56) | 0.004 |  |  |  | -1.51*  (-2.46,-0.56) | 0.004 |
| Louisiana | | 2.30(1.87,2.81) | 2.25(1.85,2.73) | 1.46(1.14,1.83) | 1.52(1.20,1.89) | 1813 | 2.03(1.94,1.94) | 2001-2012 | 0.19  (-1.66,2.08) | 0.827 | 2012-2019 | -6.46*  (-10.03,-2.75) | 0.002 | -2.45*  (-4.12,-0.74) | 0.005 |
| Maine | | 3.33(2.46,4.43) | 3.17(2.40,4.14) | 2.36(1.70,3.22) | 2.43(1.76,3.30) | 919 | 2.84(2.65,2.65) | 2001-2019 | -1.3*  (-2.24,-0.35) | 0.011 |  |  |  | -1.3*  (-2.24,-0.35) | 0.011 |
| Maryland | | 2.73(2.29,3.22) | 2.58(2.19,3.02) | 1.63(1.34,1.97) | 1.93(1.63,2.29) | 2775 | 2.4(2.31,2.31) | 2001-2011 | -0.38  (-2.54,1.84) | 0.717 | 2011-2019 | -5.45*  (-8.33,-2.47) | 0.002 | -2.66*  (-4.29,-1.01) | 0.002 |
| Massachusetts | | 2.88(2.49,3.32) | 3.02(2.64,3.44) | 2.35(2.02,2.71) | 2.20(1.89,2.54) | 3953 | 2.72(2.63,2.63) | 2001-2012 | -0.03  (-1.39,1.35) | 0.965 | 2012-2019 | -5.08*  (-7.61,-2.48) | 0.001 | -2.02*  (-3.22,-0.81) | 0.001 |
| Michigan | | 2.09(1.82,2.40) | 2.49(2.20,2.81) | 2.08(1.82,2.36) | 2.19(1.93,2.47) | 4980 | 2.33(2.27,2.27) | 2001-2011 | 1.05  (-0.32,2.44) | 0.122 | 2011-2019 | -2.41*  (-4.18,-0.61) | 0.012 | -0.5  (-1.51,0.51) | 0.33 |
| Missouri | | 3.17(2.73,3.66) | 2.98(2.58,3.42) | 2.36(2.02,2.75) | 2.54(2.18,2.93) | 3726 | 2.91(2.81,2.81) | 2001-2019 | -1.27*  (-2.03,-0.5) | 0.003 |  |  |  | -1.27*  (-2.03,-0.5) | 0.003 |
| Nevada | | 3.38(2.59,4.34) | 2.13(1.64,2.72) | 2.57(2.04,3.21) | 1.97(1.52,2.51) | 1416 | 2.75(2.61,2.61) | 2001-2019 | -1.7*  (-3.08,-0.3) | 0.021 |  |  |  | -1.7*  (-3.08,-0.3) | 0.021 |
| New Jersey | | 2.83(2.49,3.20) | 2.43(2.14,2.76) | 1.89(1.64,2.17) | 1.69(1.45,1.95) | 4539 | 2.39(2.32,2.32) | 2001-2014 | -1.25*  (-2.08,-0.4) | 0.007 | 2014-2019 | -6.99*  (-10.53,-3.31) | 0.001 | -2.88*  (-3.97,-1.77) | <0.001 |
| New York | | 2.42(2.21,2.65) | 2.23(2.03,2.43) | 1.64(1.48,1.81) | 1.46(1.31,1.62) | 8532 | 2.04(2,2) | 2001-2012 | 0.01  (-0.79,0.81) | 0.986 | 2012-2019 | -5.89*  (-7.48,-4.26) | 0 | -2.33*  (-3.06,-1.59) | <0.001 |
| North Carolina | | 3.05(2.68,3.46) | 2.68(2.38,3.01) | 2.05(1.80,2.33) | 2.26(2.00,2.54) | 5183 | 2.68(2.61,2.61) | 2001-2009 | 1.09  (-1.15,3.38) | 0.316 | 2009-2019 | -3.85*  (-5.3,-2.38) | 0 | -1.69*  (-2.85,-0.51) | 0.005 |
| Ohio | | 2.59(2.31,2.90) | 2.83(2.55,3.13) | 2.67(2.40,2.96) | 2.36(2.11,2.63) | 6830 | 2.71(2.65,2.65) | 2001-2010 | 2.37*  (0.61,4.16) | 0.012 | 2010-2019 | -2.43*  (-4.05,-0.78) | 0.007 | -0.06  (-1.16,1.05) | 0.917 |
| Oklahoma | | 3.30(2.73,3.96) | 2.72(2.23,3.28) | 2.66(2.19,3.20) | 2.26(1.84,2.76) | 2366 | 3.08(2.96,2.96) | 2001-2019 | -1.84*  (-2.62,-1.05) | 0 |  |  |  | -1.84*  (-2.62,-1.05) | <0.001 |
| Oregon | | 3.16(2.61,3.79) | 3.05(2.55,3.61) | 2.59(2.16,3.08) | 2.03(1.66,2.46) | 2450 | 2.91(2.8,2.8) | 2001-2012 | 0.29  (-1.31,1.92) | 0.706 | 2012-2019 | -5.8*  (-8.88,-2.61) | 0.002 | -2.12*  (-3.57,-0.66) | 0.005 |
| Pennsylvania | | 2.76(2.49,3.05) | 2.77(2.51,3.05) | 2.37(2.14,2.62) | 2.19(1.97,2.43) | 8104 | 2.71(2.65,2.65) | 2001-2011 | 0.58  (-0.5,1.68) | 0.267 | 2011-2019 | -3.82*  (-5.3,-2.32) | 0 | -1.4*  (-2.22,-0.57) | 0.001 |
| Rhode Island | | 4.00(2.93,5.34) | 2.38(1.60,3.43) | 2.27(1.50,3.33) | 2.42(1.68,3.43) | 642 | 2.61(2.41,2.41) | 2001-2019 | -2.16*  (-3.91,-0.37) | 0.021 |  |  |  | -2.16*  (-3.91,-0.37) | 0.021 |
| South Carolina | | 2.43(1.97, 2.96) | 2.96(2.51 ,3.47) | 2.07(1.72,2.48) | 1.9(1.57,2.27) | 2383 | 2.45(2.35,2.55) | 2001-2013 | 0.56  (-1.38,2.54) | 0.547 | 2013-2019 | -6.14*  (-10.93,-1.08) | 0.021 | -1.72  (-3.66,0.25) | 0.087 |
| Tennessee | | 2.43(2.05,2.87) | 3.33(2.92,3.79) | 2.64(2.29,3.04) | 2.66(2.31,3.05) | 3804 | 2.89(2.8,2.8) | 2001-2013 | 1.45*  (0.2,2.72) | 0.026 | 2013-2019 | -4.29*  (-7.31,-1.18) | 0.011 | -0.5  (-1.73,0.73) | 0.423 |
| Texas | | 2.68(2.45,2.93) | 2.25(2.06,2.45) | 1.81(1.65,1.98) | 1.66(1.51,1.81) | 9796 | 2.26(2.21,2.21) | 2001-2010 | -0.5  (-1.65,0.67) | 0.375 | 2010-2019 | -4.26*  (-5.36,-3.15) | 0 | -2.39*  (-3.12,-1.66) | <0.001 |
| Utah | | 3.15(2.34,4.12) | 3.51(2.78,4.35) | 2.55(1.97,3.24) | 2.77(2.18,3.48) | 1356 | 3.28(3.1,3.1) | 2001-2011 | 1.37  (-0.91,3.7) | 0.219 | 2011-2019 | -4.47*  (-7.21,-1.65) | 0.005 | -1.27  (-2.89,0.38) | 0.13 |
| Vermont | | 3.12(1.90,4.84) | 2.31(1.32,3.79) | 1.98(1.14,3.27) | 2.22(1.27,3.65) | 397 | 2.77(2.5,2.5) | 2001-2019 | -2.26*  (-3.75,-0.74) | 0.006 |  |  |  | -2.26*  (-3.75,-0.74) | 0.006 |
| Virginia | | 2.84(2.46,3.27) | 2.76(2.42,3.14) | 1.96(1.68,2.26) | 2.05(1.78,2.35) | 4228 | 2.67(2.59,2.59) | 2001-2010 | 0.57  (-1.23,2.41) | 0.508 | 2010-2019 | -4.48*  (-6.19,-2.73) | 0 | -1.98*  (-3.12,-0.83) | 0.001 |
| Washington | | 3.16(2.71,3.65) | 2.76(2.39,3.16) | 2.24(1.92,2.59) | 2.34(2.02,2.69) | 3799 | 2.8(2.71,2.71) | 2001-2014 | -0.33  (-1.35,0.71) | 0.506 | 2014-2019 | -4.95*  (-8.94,-0.78) | 0.024 | -1.63*  (-2.89,-0.36) | 0.012 |

PY, person-years; APC, annual percent changes; AAPC, average APC; CI, confidence interval; CM, Cutaneous Melanoma.

*Joint point software selected joint point with significant annual percentage changes (P < 0.05); ^a^Mortality rate data is available only for the years 2010, 2014, and 2015. ^b^Mortality rate data is available only for the years 2006, 2009, 2011, 2013, 2014, 2015, 2017 and 2019. ^c^Mortality rate records are unavailable for 2001, 2005, and 2012. Mortality trend estimates for the District of Columbia were unavailable because annual death counts were too small and were suppressed in the source database, precluding APC/AAPC estimation.
