# Supplementary material for: Geographic and population disparities in cutaneous melanoma in the United States: state-level trends and national population-level analyses
Source: BMC Public Health. 2026 May 2;26:1986. doi: 10.1186/s12889-026-27396-z (PMC13321868; doi:10.1186/s12889-026-27396-z)
Supplement: Supplementary file 3 — Supplementary Material 3. Supplementary Table 3. Multivariable linear regression models of state-level AAPC in cutaneous melanoma incidence (2001–2019), with obesity prevalence as the primary exposure. AAPC, average annual percent change in age-adjusted melanoma incidence (2001–2019). β indicates the absolute change in AAPC (percentage points per year) per unit increase in each covariate. Minor indoor tanning policy reflects state-level restrictions on indoor tanning for minors. School sunscreen policy reflects laws allowing students to carry and self-apply sunscreen at school. Non-Hispanic White (%) is derived from the 2010 U.S. Census at the state level. UV=ultraviolet. Analysis includes 49 states (Alaska and Hawaii excluded due to missing UV data). *P < 0.05. Supplementary Table 4. Multivariable linear regression models of state-level AAPC in cutaneous melanoma incidence (2001–2019), with physical activity level as the primary exposure. AAPC, average annual percent change in age-adjusted melanoma incidence (2001–2019). β indicates the absolute change in AAPC (percentage points per year) per unit increase in each covariate. Minor indoor tanning policy reflects state-level restrictions on indoor tanning for minors. School sunscreen policy reflects laws allowing students to carry and self-apply sunscreen at school. Non-Hispanic White (%) is derived from the 2010 U.S. Census at the state level. UV=ultraviolet.Analysis includes 49 states (Alaska and Hawaii excluded due to missing UV data). *P < 0.05. [file 12889_2026_27396_MOESM3_ESM.docx]

| Supplementary Table 3. Multivariable linear regression of state-level AAPC in CM (2001–2019) with obesity prevalence as the main exposure | | |
| --- | --- | --- |
| Variable | β(95%CI) | P value |
| Obesity (%) | 0.198 (0.059, 0.336) | 0.006* |
| Health care rate | 19.006 (-0.322, 38.333) | 0.054 |
| average daily solar insolation | 0.000 (-0.000, 0.001) | 0.840 |
| Personal doctor rate | -14.685 (-26.163, -3.208) | 0.013* |
| Minor indoor tanning policy | -0.009 (-0.652, 0.634) | 0.978 |
| School sunscreen policy | 1.102 (-1.891, 4.095) | 0.461 |
| non-Hispanic White (%) | 0.035 (-0.004, 0.073) | 0.074 |

AAPC, average annual percent change in age-adjusted melanoma incidence (2001–2019). β indicates the absolute change in AAPC (percentage points per year) per unit increase in each covariate. Minor indoor tanning policy reflects state-level restrictions on indoor tanning for minors. School sunscreen policy reflects laws allowing students to carry and self-apply sunscreen at school. Non-Hispanic White (%) is derived from the 2010 U.S. Census at the state level. UV=ultraviolet. Analysis includes 49 states (Alaska and Hawaii excluded due to missing UV data). *P < 0.05.

| Multivariable linear regression models of state-level AAPC in cutaneous melanoma incidence (2001–2019), with obesity prevalence as the primary exposure | | |
| --- | --- | --- |
| Variable | β(95%CI) | P value |
| Physical activity (%) | -0.156 (-0.246, -0.066]) | 0.001* |
| Health care rate | 19.09(1.14, 37.04) | 0.038* |
| average daily solar insolation | 0.000 (-0.000, 0.001) | 0.659 |
| Personal doctor rate | -14.91(-25.83, -3.996) | 0.0086* |
| Minor indoor tanning policy | 0.046 (-0.575, 0.666) | 0.883 |
| School sunscreen policy | 1.45 (-1.45, 4.35) | 0.319 |
| non-Hispanic White (%) | 0.053 (0.0161, 0.0893) | 0.006* |

AAPC, average annual percent change in age-adjusted melanoma incidence (2001–2019). β indicates the absolute change in AAPC (percentage points per year) per unit increase in each covariate. Minor indoor tanning policy reflects state-level restrictions on indoor tanning for minors. School sunscreen policy reflects laws allowing students to carry and self-apply sunscreen at school. Non-Hispanic White (%) is derived from the 2010 U.S. Census at the state level. UV=ultraviolet.Analysis includes 49 states (Alaska and Hawaii excluded due to missing UV data). *P < 0.05.
