# Supplementary figures and images for: Geographic and population disparities in cutaneous melanoma in the United States: state-level trends and national population-level analyses
Source: BMC Public Health. 2026 May 2;26:1986. doi: 10.1186/s12889-026-27396-z (PMC13321868; doi:10.1186/s12889-026-27396-z)

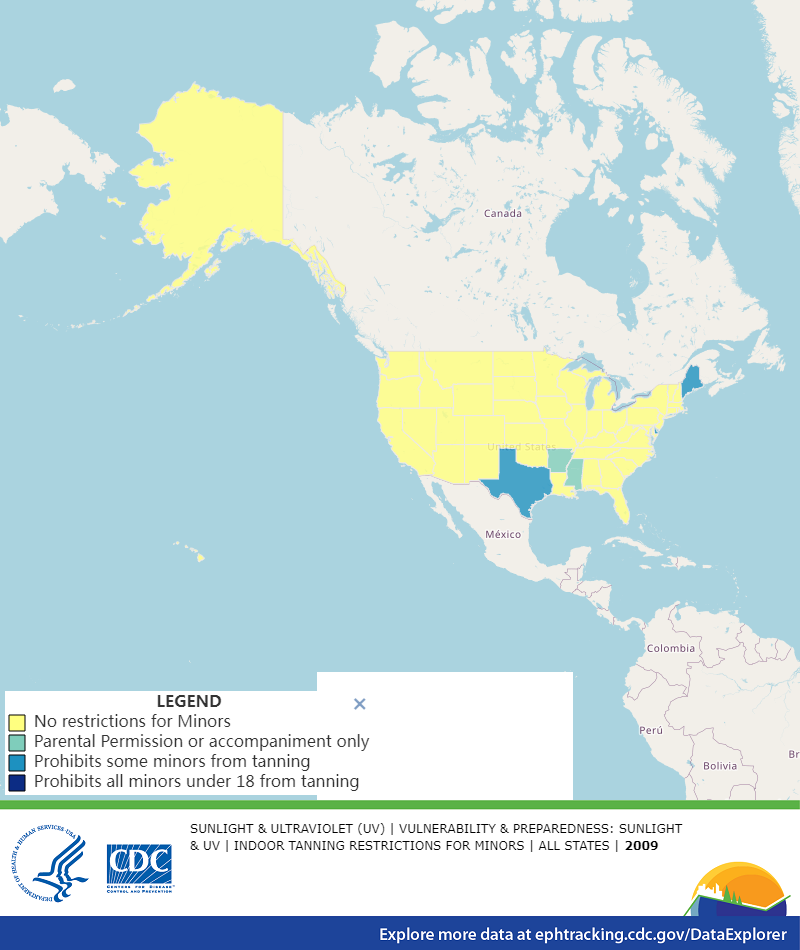

Supplement: Supplementary file 4 — Supplementary Material 4. Supplementary Figure 1: State-level legislation on indoor tanning restrictions for minors in 2009. [file 12889_2026_27396_MOESM4_ESM.jpg]

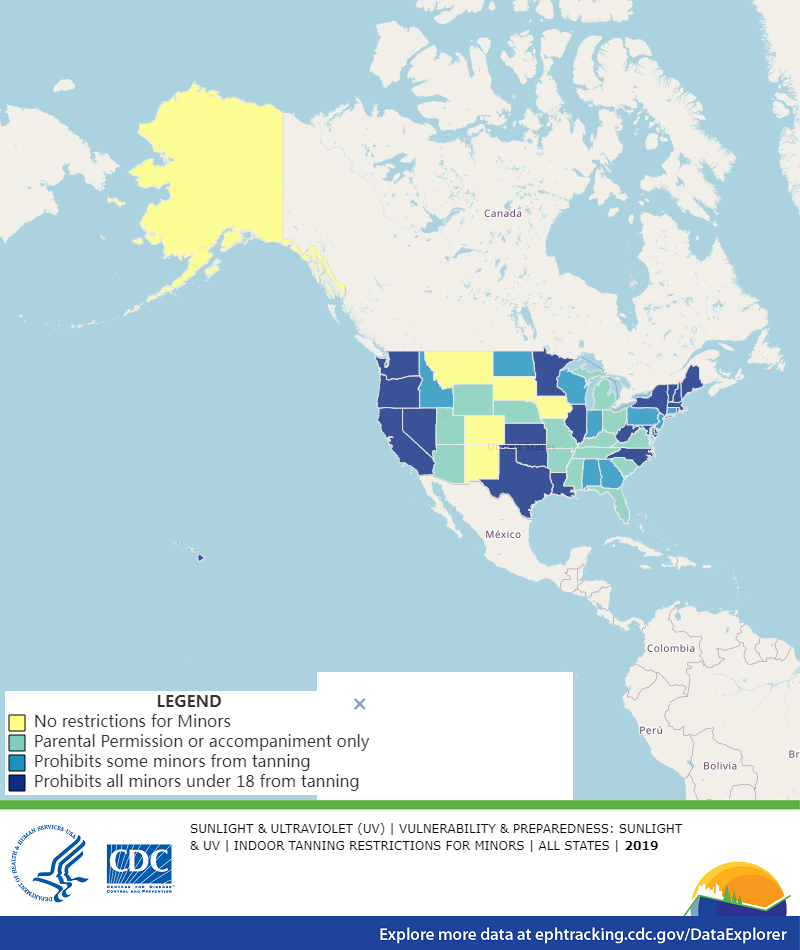

Supplement: Supplementary file 5 — Supplementary Material 5. Supplementary Figure 2: State-level legislation on indoor tanning restrictions for minors in 2019. [file 12889_2026_27396_MOESM5_ESM.jpg]

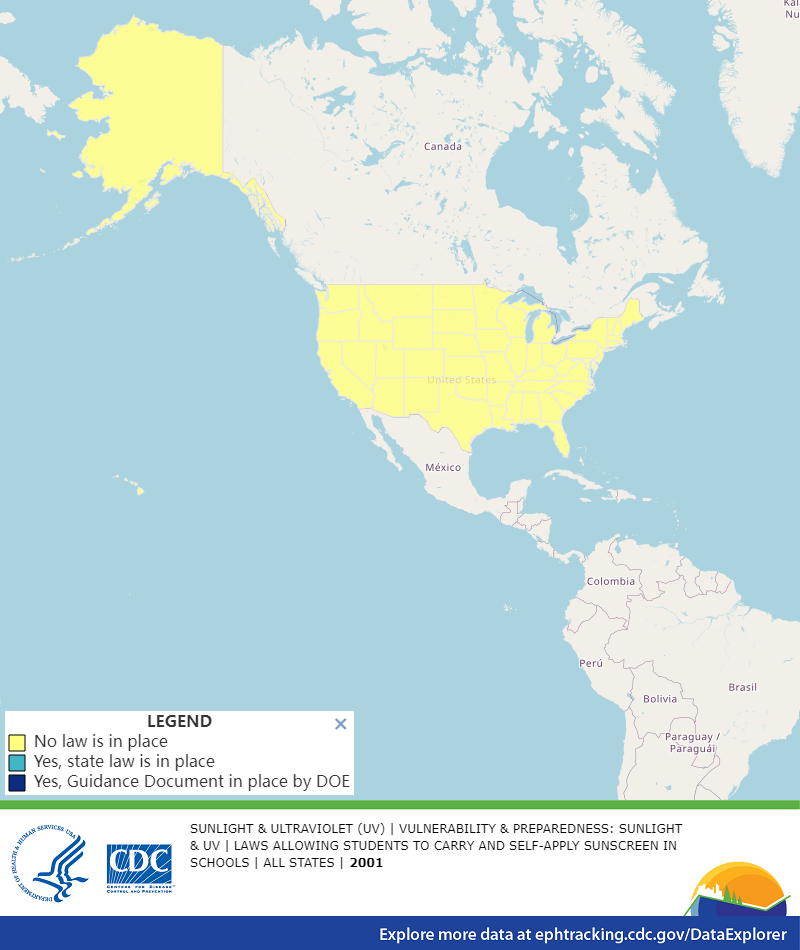

Supplement: Supplementary file 6 — Supplementary Material 6. Supplementary Figure 3: State-level laws allowing students to carry and self-apply sunscreen in schools in 2001. [file 12889_2026_27396_MOESM6_ESM.jpg]

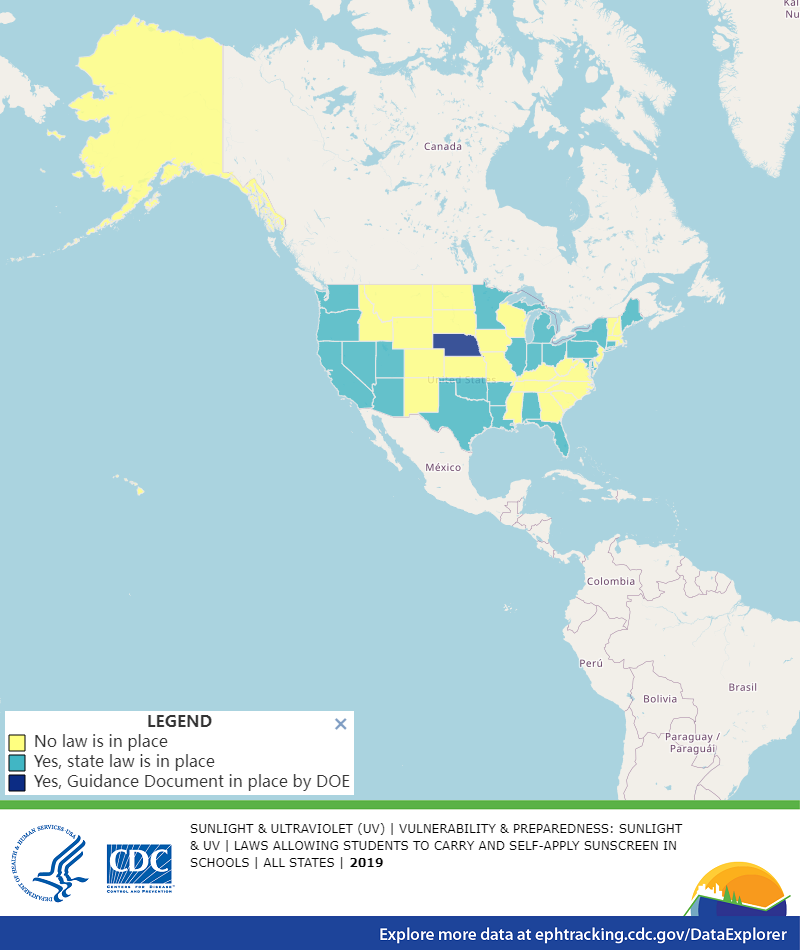

Supplement: Supplementary file 7 — Supplementary Material 7. Supplementary Figure 4: State-level laws allowing students to carry and self-apply sunscreen in schools in 2019. Supplementary Figure 1-4. State-level indoor tanning and school sunscreen policies in selected years. These policy maps were downloaded directly from the CDC's National Environmental Public Health Tracking Network. Minor indoor tanning restrictions and school sunscreen policies were included as covariates in the multivariable ecological models of state-level AAPC in CM incidence (Supplementary Tables 3 and 4). Access the data via the following URL: https://ephtracking.cdc.gov/DataExplorer/?c=19. [file 12889_2026_27396_MOESM7_ESM.jpg]

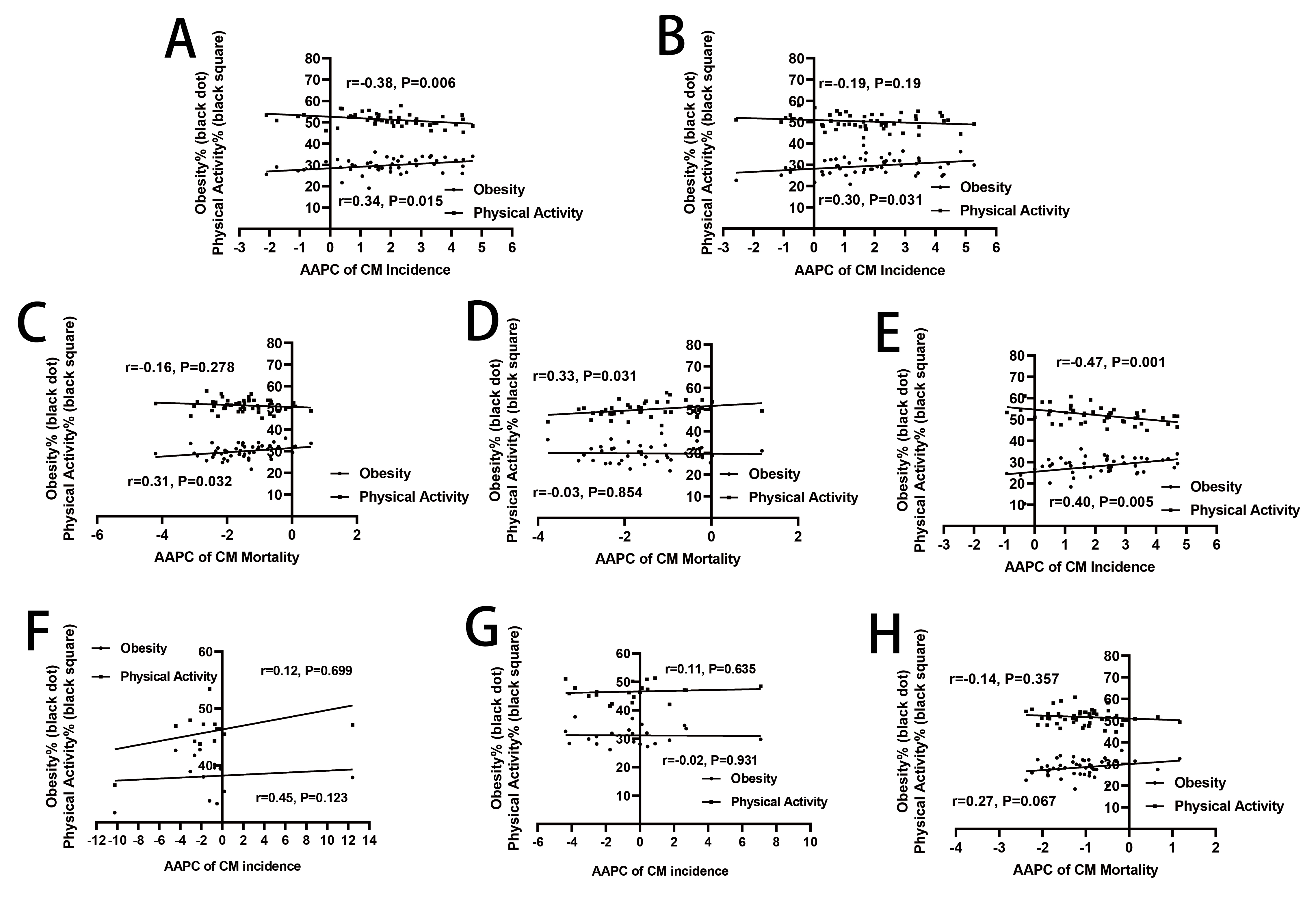

Supplement: Supplementary file 8 — Supplementary Material 8. Supplementary Figure 5: Association between state-level obesity, physical activity and average annual percent changes (AAPC) of MOTS incidence/mortality during 2001-2019, by sex and race/ethnicity. A: Obesity, Physical Activity, and AAPC of male incidence; B: Obesity, Physical Activity, and AAPC of female incidence; C: Obesity, Physical Activity, and AAPC of male mortality; D: Obesity, Physical Activity, and AAPC of female mortality; E: Obesity, Physical Activity, and AAPC of Non-Hispanic Whites incidence; F: Obesity, Physical Activity, and AAPC of Non-Hispanic Blacks incidence. G: Obesity, Physical Activity, and AAPC of Hispanic incidence; H: Obesity, Physical Activity, and AAPC of Non-Hispanic Whites mortality. The X axis denotes AAPC of CM incidence or mortality. The Y axis of the Black dot denotes average percentage of state-level obesity during 2011-2019, the Y axis of the Black square denotes average percentage of state-level physical activity during 2011-2019. Each Black dot and Black square denotes states and the District of Columbia. [file 12889_2026_27396_MOESM8_ESM.jpg]
